# Inhibition of N-acetylglucosaminyltransferase V alleviates diabetic cardiomyopathy in mice by attenuating cardiac hypertrophy and fibrosis

**DOI:** 10.1186/s12986-024-00797-w

**Published:** 2024-07-30

**Authors:** Ran Zhao, Jianqiang Hu, He Wen, Jieqiong Zhao, Ying Wang, Xiaona Niu, Mingming Zhang, Tingting Wang, Yan Li

**Affiliations:** Department of Cardiology, Tangdu Hospital, Air Force Medical University, Xinsi Road No.569, Xi’an, 710038 People’s Republic of China

**Keywords:** Diabetic cardiomyopathy, GnT-V, Integrinβ1, Hypertrophy, Fibrosis

## Abstract

**Background:**

The pathogenesis of diabetic cardiomyopathy is closely linked to abnormal glycosylation modifications. N-acetylglucosaminyltransferase V (GnT-V), which catalyzes the production of N-linked -1–6 branching of oligosaccharides, is involved in several pathophysiological mechanisms of many disorders, including cardiac hypertrophy and heart failure. However, the mechanism by which GnT-V regulates cardiac hypertrophy in diabetic cardiomyopathy is currently poorly understood. In this study, we investigated the role of GnT-V on myocardial hypertrophy in diabetic cardiomyopathy and elucidated the underlying mechanisms.

**Material and methods:**

Streptozotocin (STZ) was intraperitoneally injected into mice to induce diabetic cardiomyopathy. An adeno-associated virus (AAV) carrying negative control small hairpin RNA (shNC) or GnT-V-specifc small hairpin RNA (shGnT-V) was used to manipulate GnT-V expression. In our study, forty male C57BL/6J mice were randomly divided into four groups (10 mice per group): control mice with AAV-shNC, diabetic cardiomyopathy mice with AAV-shNC, control mice with AAV-shGnT-V, and diabetic cardiomyopathy mice with AAV-shGnT-V. In addition, H9C2 cells and primary neonatal cardiac fibroblasts treated with high glucose were used as a cell model of diabetes. Analysis of cardiac hypertrophy and fibrosis, as well as functional studies, were used to investigate the underlying molecular pathways.

**Results:**

AAV-mediated GnT-V silencing dramatically improved cardiac function and alleviated myocardial hypertrophy and fibrosis in diabetic mice. In vitro experiments demonstrated that GnT-V was elevated in cardiomyocytes and induced cardiomyocyte hypertrophy in response to high glucose stimulation. GnT-V knockdown significantly reduced the expression of the integrinβ1 signaling pathway, as evidenced by decreased downstream ERK1/2 activity, which inhibited cardiomyocyte hypertrophy accompanied by reduced ANP, BNP, and β-MHC expression. Furthermore, knocking down GnT-V expression lowered the TGF-β1-Smads signaling pathway, which reduced the expression of α-SMA, collagen I, and collagen III.

**Conclusions:**

Overall, our research indicated that GnT-V may be a useful therapeutic target to treat diabetic cardiomyopathy, primarily in the inhibition of myocardial hypertrophy and fibrosis.

**Supplementary Information:**

The online version contains supplementary material available at 10.1186/s12986-024-00797-w.

## Introduction

Diabetes mellitus with its rapidly increasing incidence and prevalence has become a markedly independent risk factor for heart failure [1]. Diabetic cardiomyopathy is a specific type of diabetic cardiac complication characterized by structural and functional abnormalities such as left ventricular dysfunction, myocardial hypertrophy, cardiac fibrosis in the absence of any coronary artery disease, and other cardiac pathologies [2]. In the past, researchers observed multiple mechanisms that could affect the pathogenesis of diabetic cardiomyopathy, such as inflammation, oxidative/ nitrosative stress, fibrosis, and mitochondrial dysfunction [3–5]. However, the molecular and cellular mechanisms of diabetic cardiomyopathy are not thoroughly understood and the outcomes of current therapeutic strategies are unsatisfactory.

Myocardial hypertrophy and fibrosis contribute to the changes in pathological function and structure in diabetic cardiomyopathy [6]. Pathological hypertrophy is characterized by the reorganization of contractile proteins, activation of the fetal gene program, increased cell size, and cytoskeletal remodeling under hyperglycemic conditions [7]. Several studies showed that integrins and integrin-associated signaling pathways were involved in heart growth and pathological changes. Pathological cardiac hypertrophy was accompanied by an increase in β_1_ concentrations and redistribution of the β_3_ integrin molecules [8, 9]. In particular, the deficiency of β_1_ integrin led to hypertrophic changes in company with reduced basal contractility and relaxation, which significantly promoted myocardial dysfunction after myocardial infarction [10]. However, the actual role played by the β_1_ integrin in diabetes cardiomyopathy-related cardiac hypertrophy is still unknown.

Glycosylation is a common type of post-translational modification, which is exerted by the glycosyltransferases and glycosidases. Aberrant glycosylation has been associated with disease progression and cellular carcinogenesis,[11, 12]. Recently, N-linked glycosylation has been regarded as an important indicator for assessing the endocrine and metabolic status of diabetic patients, and most of the diabetes-associated N-glycoprotein alterations are due to aberrant expression of glycosyltransferase genes, such as N-acetylglucosaminyltransferase V (GnT-V, also known as Mgat5) [13–15]. GnT-V facilitates the transfer of β1,6-linked GlcNAc units from UDP-GlcNAc to the α1,6-linked mannose present on the trimannosyl core of N-linked glycans [16]. Previous studies have shown that GnT-V modifies signaling pathway receptor-associated N-glycans, such as epidermal growth factor (EGF) and platelet-derived growth factor (PDGF) to regulate disease processes [17, 18]. Furthermore, in our previous study, we found that GnT-V promotes diabetic cardiac fibrosis by mediating CD147 glycosylation [19]. However, whether GnT-V regulates ventricular hypertrophy in diabetic cardiomyopathy is unclear.

Thus, the goal of this investigation was to look into the potential effects of GnT-V in diabetic cardiomyopathy, namely cardiac hypertrophy, as well as the underlying in vivo and in vitro molecular pathways.

## Material and methods

### Animals

The Air Force Medical University Committee on Animal Care has assessed and authorized all animal procedures, complying with the Institutional Guidelines for Animal Research and the Guide for the Care and Use of Laboratory Animals (2011).

### Treatment and animal models

We purchased male C57BL/6J mice from the Experimental Animal Center at the Air Force Medical University, aged 6–8 weeks. After a week of acclimation, the mice were randomly grouped: (1) control + AAV-shNC group; (2) diabetic cardiomyopathy + AAV-shNC group (DCM + AAV-shNC); (3) control + AAV-shGnT-V group; (4) diabetic cardiomyopathy + AAV-shGnT-V group (DCM + AAV-shGnT-V). Diabetic mice were injected intraperitoneally with streptozotocin (STZ) (50 mg/kg in citrate buffer, pH = 4.5) for five days, while control mice received the same amount of citrate buffer as described above [21]. After seven days, diabetic animals were deemed successful as their tail vein's randomly measured blood glucose level was 16.7 mmol/L, using a Contour glucose meter manufactured by Roche in Germany. GenePharma Company (Shanghai, China) provided the AAV vector for the shRNA-mediated knockdown of GnT-V and the control virus. The following shRNA sequence was used to target GnT-V: 5'-GGAATCAGGCTTCAAGATTGC-3'. The shRNA scrambled sequences (shNC) were created as follows: 5'-ACTACCGTTGTTATAGGTG-3'. After four weeks of STZ, 40 μl shRNA or GnT-V shRNA was injected into three LV myocardium locations. All mice underwent a 12-h light cycle followed by a 12-h dark cycle at a room temperature of 25 °C for 12 weeks. Unrestricted access to food and water was provided to all the animals.

### Echocardiography

As previously described [20], transthoracic echocardiography using a Vevo-3100 echocardiography equipment (Visual Sonics Inc., Canada) was performed in M-mode. Mice were anesthetized with 1.0% isoflurane in oxygen, with heart rates maintained at 400–500 beats per minute, and placed on a warming pad. A 30 MHz linear transducer was used to measure the internal dimension of the left ventricle in diastole (LVIDd) and systole (LVIDs). In addition, computer methods were used to calculate the interventricular septal thickness in systole and diastole, the left ventricular ejection fraction (LVEF), the left ventricular fractional shortening (LVFS) and the left ventricular mass (LV mass). All measures were taken during six consecutive cardiac cycles by a blinded investigator.

### Hematoxylin and eosin (HE) and Masson staining

Mouse hearts were fixed in 4% paraformaldehyde and embedded in paraffin. The hearts were cut into 5-μm thick slices. For cell morphology, the slices were stained with HE, and the cardiac collagen content was determined using Masson. Images were captured using an Olympus IX71 fluorescent microscope (Japan).

### Immunohistochemistry (IHC)

The heart tissues of mice were fixed in paraffin after being treated overnight with 4% (wt/vol) paraformaldehyde. Subsequently, the tissues were sliced into 5 μm-thick sections. The sections were incubated overnight at 4°C with a primary GnT-V antibody (Abcam, ab87977, 1:150) and subsequently stained. Next, the sections were incubated for 40 min with a biotinylated anti-mouse IgG horseradish peroxidase (HRP)-conjugated secondary antibody (Proteintech, SA00001-1, 1:200) at room temperature. Using diaminobenzide to view the slides, images were taken with a fluorescence microscope (Nikon 80i, Tochigi, Japan).

### Wheat germ agglutinin (WGA) staining

As previously mentioned [21], wheat germ agglutinin (WGA) staining was used to quantify cell size. In brief, images were captured employing an Olympus FV1000 confocal laser scanning microscope. In five successive mouse slices, the myocytes were traced, and ImageJ was used to calculate the diameter of the myocytes.

### Treatment and cell culture

The H9C2 cells were acquired from the American Type Culture Collection located in Manassas, Virginia. In brief, H9C2 cells were cultured in Dulbecco's modified Eagle's medium (DMEM, Hyclone) supplemented with 10% fetal bovine serum (FBS, Gibco). Penicillin and streptomycin were added to the cells at a ratio of 1:100, after which they were incubated at 37^。^C and 5% CO2. Cells were processed for the experiments when they were 70%–80% confluent.

Primary cardiac fibroblasts were isolated from the neonatal rats (within one-three days of their birth), as described previously [19]. Briefly, twenty or more hearts from neonatal rats were cut into samll sections and placed together in collagenase type II (1 mg/mL, Thermo Fisher Scientific, Waltham, MA, USA). Pooled cell suspensions were centrifuged and resuspended in Dulbecco's modified Eagle's medium (DMEM, Hyclone) supplemented with 10% fetal bovine serum (FBS, Gibco), 100 U/ml penicillin and 100 μg/ml streptomycin. After removing larger pieces of tissue using a 150 mesh screen, the suspension was incubated in culture flasks for 1.5 h, which makes fibroblasts preferentially adhere to the bottom of the culture bottles. Non-adherent cells were removed and the medium was changed. The primary cardiac fibroblasts were incubated for 24 h in three different glucose concentrations: normal (5.5 mmol/L), moderate (25 mmol/L), and high (33 mmol/L).

Adenoviral vectors containing shGnT-V (Ad-shGnT-V) and adenoviral vectors corresponding to negative controls (Ad-shNC) were designed and produced by GenePharma Company (Shanghai, China). In this study, the titer of the adenoviruses used was about 1.2*10^10^ PFU/ml. The sequences were: Ad-shGnT-V: 5'-GGATGATGCTTCTACACTTCA-3'; Ad-shNC: 5'-ACTACCGTTGTTATAGGTG-3'. Utilizing BTT-3033 (10 mol/L; Tocris Bioscience), integrin-β1 was inhibited. The plasmid that expresses integrin-β1 was purchased from Hanbio Biotechnology (Shanghai, China). DNA sequencing verified the construct. According to the manufacturer's instructions, Lipofectamine 3000 (Thermo Fisher Scientific, L3000001) was used to transfect the PCDNA3.1 integrinβ1-expressing plasmid. A PCDNA3.1–3 × flag served as the study's adverse control. The cells were handled in a growth-friendly environment.

### Western blot analysis

Myocardial tissues and cultured cells were collected, and the total protein was lysed by radio-immunoprecipitation assay (RIPA) bufer (P0013B, Beyotime) containing 1% phenylmethanesulfonyl fluoride (PMSF) protease inhibitor (ST506-2, Beyotime). Subsequently, the proteins were separated on SDS-PAGE gel, followed by protein transfer onto polyvinylidene difuoride (PVDF) membranes. After that, the membranes was blocked with 5% milk solution and the specifc primary antibody: GnT-V (Abcam, ab87977, 1:500), integrinβ1 (Abcam, ab183666, 1:1000), ANP (Proteintech, 27,426–1-AP, 1:1000), ERK1/2 (Cell Signaling Technology, 11,257–1-AP, 1:1000), phospho-ERK1/2 (Cell Signaling, 28,733–1-AP, 1:1000), collagen I (Abcam, ab260043, 1:2000), collagen III (Abcam, ab184993, 1:800) and β-actin (Proteintech, 81,115–1-RR, 1:1000) was incubated overnight at 4^。^C. Secondary antibodies were incubated at 4^。^C for 2 h. The GelDox XR System (Bio-Rad, CA, USA) was used to view the bands. We used Image Lab 4.0 to quantify the band intensity, and we examined three samples from each group.

### Lectin blot analysis

The lectin blot analysis was implemented to detect the expression of β1,6-GlcNAc-branches. The protein was extracted from different groups of H9C2 cells treated with normal-glucose medium or high-glucose medium. The protein lysates were electrophoresed by 10% SDS-PAGE according to the protocol of the Western blot analysis. After blocking with 5% bovine serum albumin in PBS containing 0.5% (w/v) Tween 20, the PVDF membrane (0.45µm,) was incubated with 15 μg/mL biotinylated L-PHA (Vector Laboratories, Burlingame, CA) at room temperature for 2 h. After staining with avidin-HRP, the expression of the bands was visualized by enhanced chemiluminescent (ECL). β-actin was used as the standard.

### L-PHA precipitation

The different groups of H9C2 cells were treated with normal-glucose medium or high-glucose medium. For lectin precipitation of the integrinβ1, the cell lysate (800 μg) in RIPA buffer was added to 50 μL of agarose-bound L-PHA lectin (Vector Laboratories, Burlingame, CA), and was incubated overnight at 4°C under sustaining agitation. The beads after being collected by centrifugation (5 s in a microcentrifuge at 12,000 rpm) were washed three times with ice-cold PBS, then were boiled with 5 × SDS-PAGE protein buffer at 100°C for 8 min. In virtue of SDS-PAGE and membrane transfer, N-linked β-1,6-branching on the integrinβ1 was detected with an antibody against integrinβ1.

### Immunofluorescence staining

Cardiac fibroblasts or GnT-V-deficient cardiac fibroblasts were treated using either normal-glucose medium (5.5 mmol/L) or high-glucose medium (33 mmol/L) while they were cultured on coverslips. The cells were fixed with 4% paraformaldehyde for 20 min at a temperature of 4°C. Next, to block the membrane, 3% BSA (Beyotime, Shanghai, China) was used for 2 h at room temperature. Then, to determine the α-SMA (Proteintech, 14,395–1-AP, 1:150), Vimentin (Proteintech, 10,366–1-AP, 1:200), MYH11 (Servicebio, GB151220-100, 1:200) or biotinylated phaseolus vulgaris leucoagglutinin (L-PHA) (ACMEC, S21290, 1:200) antigens, the primary antibodies were left to incubate at 4°C overnight. The second antibody (Abcam, ab6721, and ab6885, 1:100,) labeled with Alexa Fluor 488 or 594 was incubated at room temperature for 1 h. After 10 min, the nucleus was counterstained with DAPI (Beyotime, C1002, 1:1000). The image was captured from consecutive optical sections acquired at 0.5-min intervals, employing a confocal laser scanning microscopy setup (Leica, SP5-II).

### RNA extraction and RT-PCR analysis

We extracted total RNA from various cell groups using Trizol (Invitrogen, USA). The PrimeScript TM RT reagent kit (Takara) was utilized to generate cDNA, following the manufacturer's protocol. The primer sequences are as follows: GnT-V-rat F: CCCTGGAAGTTGTCCTCTCA, R: TCCTCTGCCAGTGCCTTAAT; ANP-rat F: AGTGCGGTGTCCAACACAGA, R: TCATCTTCTACCGGCATCTTCTC; BNP-rat F: TTCCAAGATGGCACATAGTTCAA, R: AGCCAGGAGGTCTTCCTAAAACA; β-MHC-rat F: CAGGGTGTTGACCTTGTCCT, R: TGATCTGGAGCTGACACTGG; β-actin-rat F: CCCGCGGAGTACAACCTTCT, R: CGTCATCCATGGCGAACT; GnT-V-mouse F: TACGGGAGCAGATCCTTGAC, R: TGACCAGATTGTCCACCTTTGA; ANP-mouse F: AATTTGCTGGACCATTTGGA, R: GCTTCTTCATTCGGCTCACT; BNP-mouse F: AAGATGGTGCAAGGGTCTG, R: TGTGGAATCAGAAGCAGGTG; β-actin-mouse F: GTCCCTCACCCTCCCAAAAG, R: GCTGCCTCAACACCTCAACC.

### Statistical analysis

Graphpad Prism 7.0 was used to analyze the data, and the results are shown as mean ± SD. To evaluate statistical differences, an unpaired Student’s *t* test was performed between two groups, and an one-way ANOVA was carried out followed by the Bonferroni post-hoc test for multiple groups. Statistical significance was considered at *p* < 0.05.

## Results

### GnT-V expression was upregulated in cardiac tissue samples acquired from diabetic mice

**Fig. 1:**
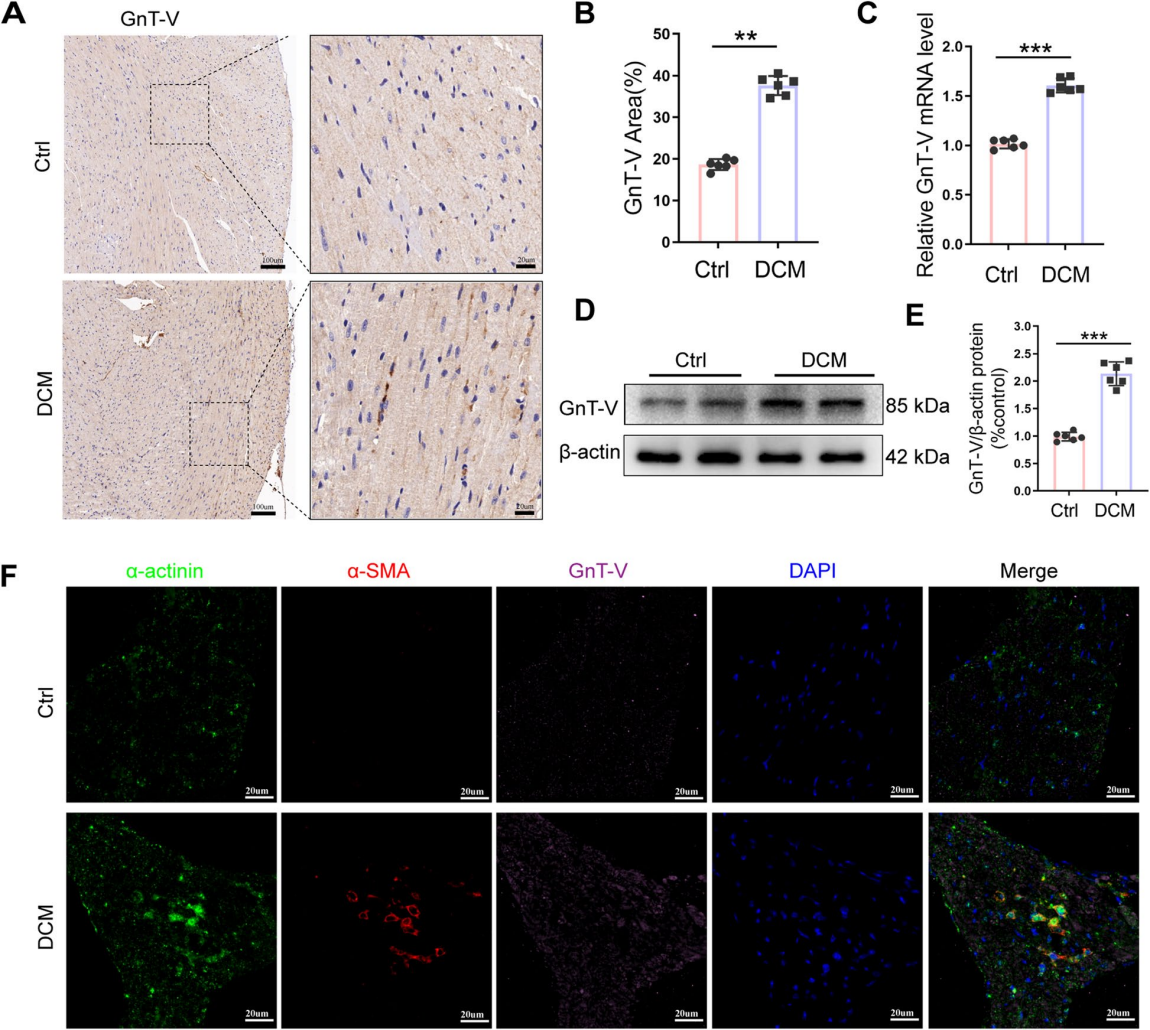
Mice with diabetes have higher levels of GnT-V expression. The level of GnT-V expression in the cardiac tissues of Ctrl and DCM mice is shown in image **A**, lift scale bar = 100 um; right scale bar = 20 m. **B** Analysis of the GnT-V (brown) expression in cardiac tissue using a statistical method. **C** GnT-V mRNA analysis in left ventricles. **D**, **E**. Exemplary immunoblots of GnT-V expression in diabetic hearts and analysis demonstrating the statistically significant variation in GnT-V levels between each group.** F** DAPI (blue), GnT-V (pink), α-SMA (red), and α-actinin (green) immunofluorescence images of the cardiac tissues of Ctrl and DCM mice, scale bar = 20 μm. Data presented as mean ± SD (*n* = 6 each group). *p < 0.05, ** < 0.01, ***p < 0.001

To evaluate the status of GnT-V in the cardiac tissue samples acquired from the diabetic mice, we initially designed a diabetic model by injecting the animals with STZ intraperitoneally for 5 consecutive days. The results of the immunohistochemical analysis revealed that the GnT-V expression levels were significantly elevated after STZ injection in comparison to the control cardiac tissue samples (Fig. 1A, B). Similarly, qRT-PCR results suggested an increase in the GnT-V mRNA levels in the left ventricular tissue samples after STZ injection (Fig. 1C). The Western blot analysis revealed increased GnT-V expression in the left ventricular tissue samples of diabetic mice as compared to the control group (Fig. 1D, E). We further carried out triple immunofluorescence staining experiments for determining the levels of α-actinin, α‐ SMA, and GnT-V in the diabetic and control tissues to measure the GnT-V expression levels in various cardiac cell populations during diabetic cardiomyopathy (Fig. 1F). The expression levels of GnT-V in cardiomyocytes and fibroblasts were significantly higher in diabetic cardiac tissues. According to these findings, GnT-V may have contributed to diabetic cardiomyopathy.

### GnT-V knockdown ameliorated diabetes-induced myocardial dysfunction and hypertrophy

**Fig. 2:**
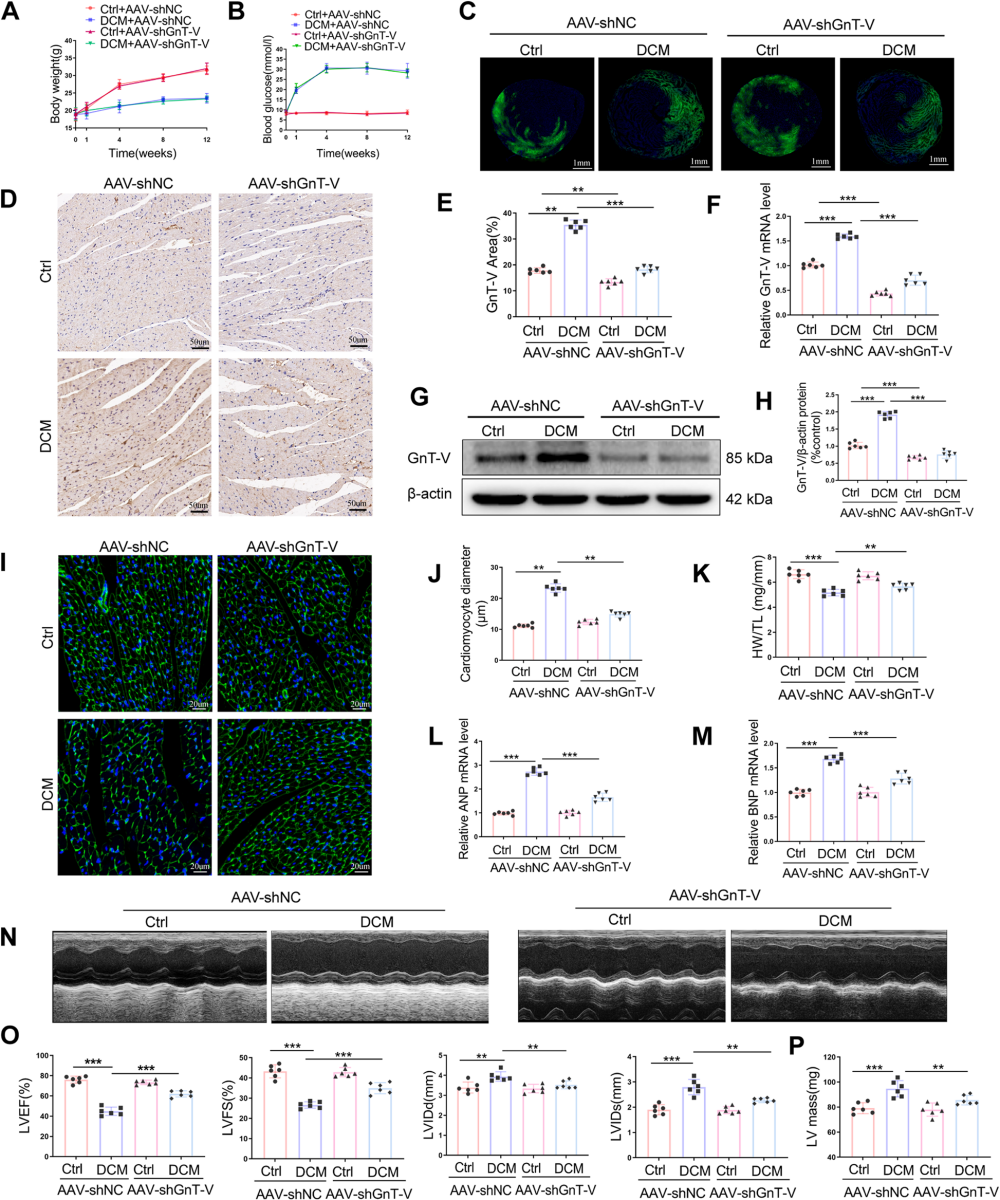
In diabetic mice, heart hypertrophy and dysfunction were prevented by GnT-V knockdown. **A** Levels of blood sugar in the various groups. **B** Mouse body weights in the various groups. **C** Typical frozen section images of isolated hearts from rats that had either shGnT-V or scramble shRNA injected into them. **D** Scale bar: 50 m. Exemplary immunohistochemistry staining images of GnT-V (brown) expression in each group's cardiac tissues. **E** GnT-V (brown) expression in cardiac tissue was analyzed quantitatively.** F** GnT-V mRNA in left ventricles was analyzed.** G**, **H**. Typical immunoblots of GnT-V expression in the walls of the left ventricle and analysis to demonstrate the significant difference in GnT-V levels between each group. **I** Typical examples of WGA staining. Cardiomyocyte outlines are visible in green, while nuclei (DAPI positive) are visible in blue fluorescence. **J** Cardiomyocyte diameter in cardiac tissue was measured quantitatively. **K** Evaluation of heart weight to tibia length (HW/TL) ratio in mice. Evaluation of the mRNA levels of ANP (**L**) and f BNP (**M**) in the myocyte cross-sectional region. **N** Left ventricular chambers as seen in representative echocardiogram. **O** EF%, FS%, LVIDs, and LVIDd of the left ventricle are evaluated. **P** LV mass is evaluated. Data presented as mean ± SD (*n* = 6 each group). *p < 0.05, ** < 0.01, ***p < 0.001

Left ventricular hypertrophy and cardiac dysfunction were seen to be prominent features of diabetic cardiomyopathy cardiac tissues. AAV vectors containing either scramble shRNA or shRNA against GnT-V were administered into the left ventricle, followed by intraperitoneal injection of STZ to assess the role of GnT-V in controlling diabetic cardiomyopathy. The GFP expression levels were highlighted in the autofluorescence photos taken from the frozen sections in the 4 groups of mice, demonstrating that the shRNA gene was successfully delivered to the myocardium by the AAV vector (Fig. 2C). The consistent changes in body weight and blood glucose levels observed in diabetic mice induced by STZ match previously reported findings (Fig. 2A, B) [22, 23]. The results of the immunohistochemistry analysis (Fig. 2D, E), qRT-PCR (Fig. 2F), and western blot analyses (Fig. 2G, H) demonstrated that following the introduction of scrambling shRNA, GnT-V expression was elevated in the left ventricular tissues of STZ-induced diabetic mice. However, treatment with GnT-V-shRNA significantly reduced the expression levels of GnT-V induced by STZ. This improved the resistance to cardiac hypertrophic responses, which was evident from the reduced cardiomyocyte size and a lower left ventricular mass (LV mass) compared to those displayed by the AAV-shNC + DCM group (Fig. 2I, J, P). Additionally, GnT-V-shRNA treatment led to a significant decrease in cardiac hypertrophy markers such as ANP and BNP (Fig. 2L, M). Echocardiography analysis showed that the control animals in the scramble shRNA or GnT-V-shRNA groups exhibited similar cardiac function measurements (Fig. 2N). Additionally, the STZ-treated mice in the GnT-V-shRNA groups demonstrated noticeably enhanced cardiac contractile performance as compared to the STZ-treated mice in the scramble shRNA group, as shown by the raised LVEF% and LVFS% values, along with additional metrics like the decreased LVIDs and LVIDd and a higher heart weight to tibia length ratio (HW/TL) (Fig. 2K, O). All of the data demonstrated that GnT-V knockdown successfully reduced diabetes-induced hypertrophy as well as myocardial dysfunction.

### GnT-V knockdown reduced fibrosis and pathologic structural abnormality in diabetic myocardium

**Fig.3:**
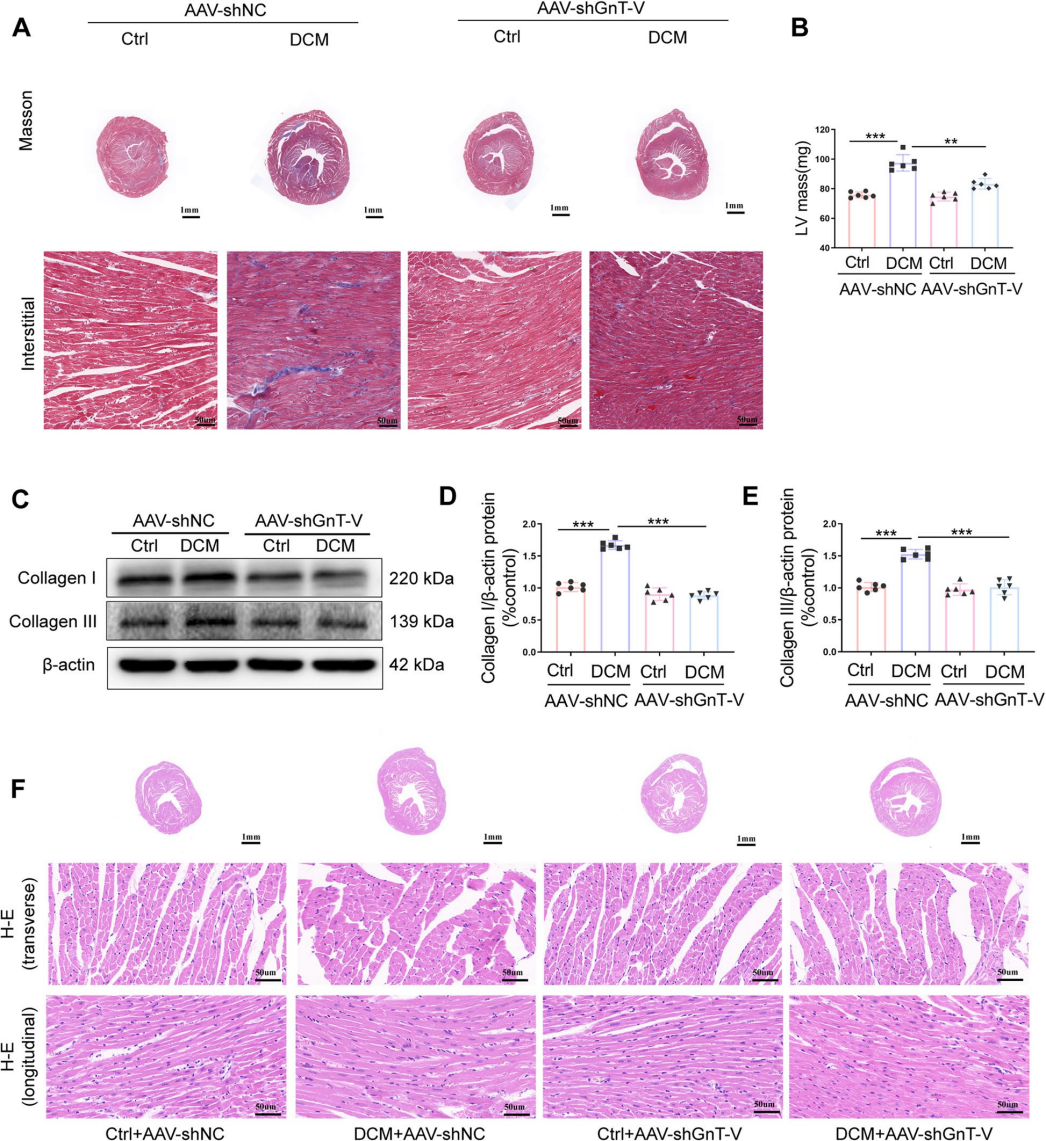
The pathogenic fibrosis in perivascular and interstitial areas as well as the pathogenic structural abnormalities in diabetic cardiomyopathy were reduced by GnT-V knockdown. **A** Pictures that perfectly capture the Masson's trichrome staining. Interstitial fibrosis (**B**) quantitative analyses. **C-E** Western blot representations of collagen I and collagen III. Collagen I and III protein expression was quantitatively analyzed. **F** HE staining showing the transverse (scale bar = 1 mm), transverse and longitudinal (scale bar = 50 mm) area of the heart tissue. Data presented as mean ± SD (*n* = 6 each group). *p < 0.05, ** < 0.01, ***p < 0.001

Given that cardiac fibrosis has a significant role in causing structural changes and myocardial remodeling in diabetic cardiomyopathy, we researched the impact of GnT-V on this condition. Masson staining procedures were used to detect collagen deposition levels in the cardiac tissue samples collected from different treatment groups. The example Masson staining images revealed that the gross morphology of hearts and interstitial collagen deposition levels in the diabetic cardiac tissues had significantly increased (Fig. 3A, B). In comparison to the scramble shRNA-injected diabetic cardiac tissues, GnT-V-shRNA treatment significantly reduced collagen accumulation. Furthermore, Western Blot data was used to assess the influence of GnT-V on cardiac fibrosis by detecting levels of collagen I and III deposition, which aligned with Masson staining results (Fig. 3C-E). The HE staining for the gross morphology of hearts further validated the effect of GnT-V in a left ventricular remodeling vision. In comparison to the Ctrl + AAV-shNC group, the longitudinal sections of the DCM + AAV-shNC group showed significant myocardial structural changes and remodeling, as shown by the hypertrophic heart and the cardiomyocytes' disorganized arrangement (Fig. 3F). Additionally, significant improvements were observed in the pathological characteristics displayed by the left ventricular samples acquired from the DCM + AAV-shGnT-V group following GnT-V knockdown. Thus, all the above results suggested that GnT-V knockdown decreased diabetes-induced myocardial fibrosis and myocardial remodeling.

### GnT-V expression was increased in HG-treated H9C2 cells

**Fig. 4:**
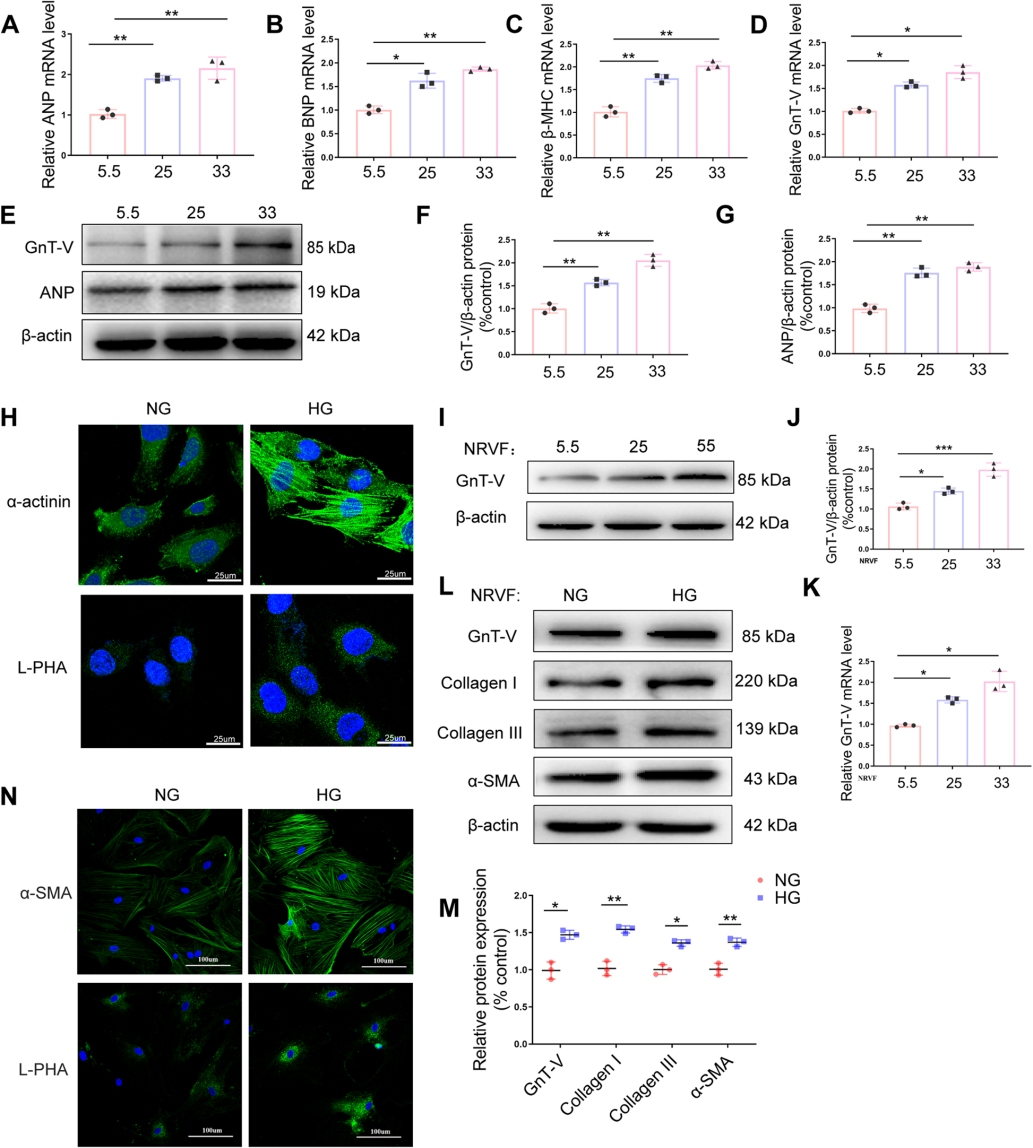
GnT-V expression was enhanced in HG-treated cardiomyocytes and GnT-V activation and fibrosis were present in HG-treated cardiac fibroblasts. For 24 h, H9C2 cells were stimulated with glucose at 5.5, 25, or 33 mM. The mRNA expression of ANP, BNP, β-MHC, and GnT-V (**A-D**) was investigated. **E** Representative immunoblots showing GnT-V and ANP expression. GnT-V (**F**) and ANP (**G**) protein expression was quantified. **H** Confocal pictures of H9C2 cells treated for 24 h with NG (5.5 mM glucose) and HG (33 mM glucose). Positive immunoreactions were shown by green indications. The expression of a-actinin is shown in the upper panels. The L-PHA expression is shown in the lower panels (scale bar = 25 μm). Cardiac fibroblasts from newborn SD rats were treated for 24 h with 5.5 mmol/L glucose (NG), 25 mmol/L glucose, or 33 mmol/L glucose (HG). **I** Representative immunoblots of GnT-V expression. **J** Quantitative study of GnT-V protein expression. **K** GnT-V expression levels were measured using qRT-PCR. **L**, **M** Representative immunoblots and quantitative analysis of GnT-V, collagens I, III, and α-SMA protein expression. Confocal pictures of cardiac fibroblasts treated with NG or HG for 24 h. Positive immunoreactions were shown by green indications. The α-SMA expression is shown in the upper panels. The L-PHA expression (**N**) is shown in the lower panels (scale bar = 100 μm). Data presented as mean ± SD. *p < 0.05, ** < 0.01, ***p < 0.001

The in vitro effect of GnT-V on cardiac hypertrophy was further assessed in this study. For this purpose, the rat heart-derived myoblastic cell line, H9C2, was employed to carry out qRT-PCR, immunoblotting, and confocal microscopy analysis. As shown in the figure (Fig. 4A-D), cardiac cells were cultured with different glucose concentrations (5.5, 25, or 33 mmol/L) for 24 h, cardiomyocyte hypertrophy was induced as indicated by the increased ANP, BNP, β-MHC, and sarcomeric a-actinin expression levels in high-glucose (HG)-treated group in comparison to the normal-glucose (NG)-treated group. Consistently, HG administration led to a considerable concentration-dependent uptick in GnT-V expression (Fig. 4E-G). Moreover, the biotinylated L-PHA, which is a product of the selective maturation of GnT-V activity, can be used as a probe for GnT-V-modified glycans. Additionally, this was found to be more concentrated in the cells treated with HG as compared to those cells that were treated with NG (Fig. 4H).

### GnT-V and fibrosis were activated in HG-treated cardiac fibroblasts

In order to reinforce the impact of GnT-V on fibrosis, we conducted in vitro experiments. For this purpose, the cardiac fibroblasts were acquired from the neonatal rats and were incubated with glucose for 24 h in a concentration-dependent manner. First, vimentin was used as a marker for CFs. We confirmed that almost all isolated cells expressed vimentin, indicating that only CFs were present (Additional file 1: Fig. S1). The Western blotting and qRT-PCR experiments revealed that the levels of GnT-V were significantly higher in the cardiac fibroblasts treated with HG than in those treated with NG (Fig. 4I-K). Meanwhile, immunofluorescence staining showed that the expression of L-PHA increased in cardiac fibroblasts treated with HG. (Fig. 4N). Immunofluorescence and western blot analysis demonstrated elevated levels of α-SMA, collagen I, and collagen III expression in the cardiac fibroblasts treated with HG, which correlates with the in vivo findings (Fig. 4L, M).

### GnT-V knockdown inhibited cardiomyocyte hypertrophy in an integrin β1- ERK1/2 dependent manner

**Fig. 5:**
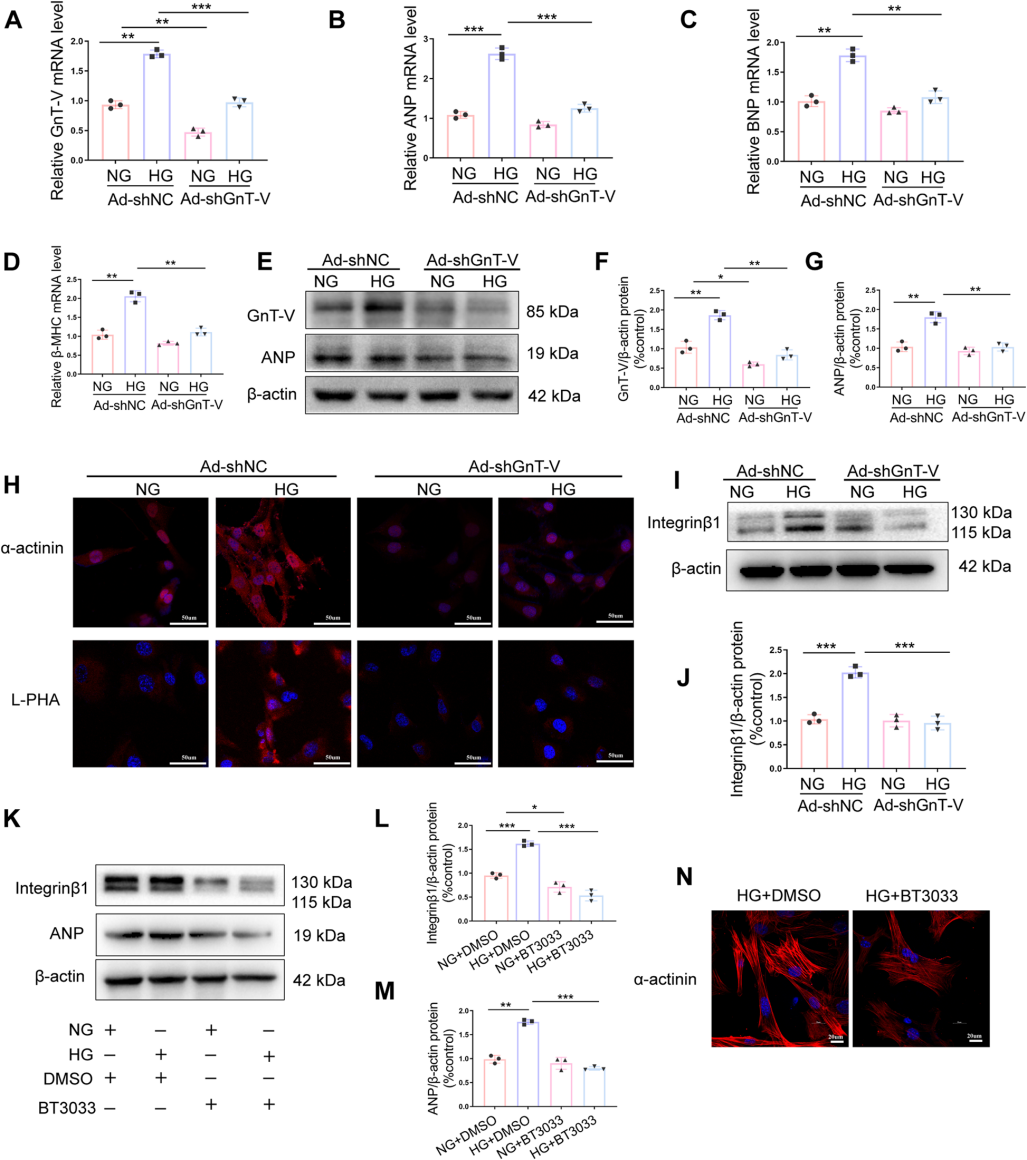
By activating integrinβ1, GnT-V enhanced cardiomyocyte hypertrophy. H9C2 cells treated with HG were transfected with shRNA against GnT-V and a negative control. GnT-V (**A**), ANP (**B**), BNP (**C**), and β-MHC (**D**) mRNA levels were examined. **E**, **F**, **G** Representative immunoblots and quantitative analysis of GnT-V and ANP protein expression. **H** Immunofluorescence was used to evaluate the amounts of α-actinin and L-PHA expression. **I**, **J** Representative immunoblots and quantitative investigation of integrin1 protein expression. **K**, **L**, **M** The immunoblots and quantitative analysis of integrinβ1 and ANP in H9C2 cells treated or not with BT-3033 (10 lmol/L; Tocris Bioscience). **N** Confocal pictures of -actinin in HG-treated H9C2 cells stimulated or not with BT-3033 (10 lmol/L; Tocris Bioscience). Data presented as mean ± SD. *p < 0.05, ** < 0.01, ***p < 0.001

To investigate the processes underlying the effect of GnT-V knockdown on cardiomyocyte hypertrophy in HG circumstances, we additionally introduced the shRNA against GnT-V into the grown H9C2 cells. As depicted in Fig. 5A, E, F, and H in H9C2 cells treated with HG, the transfection of shRNA GnT-V dramatically decreased GnT-V expression levels and inhibited the expression of β-1,6 branches, compared with the transfection of shRNA NC, as indicated by the findings of the qRT-PCR, western blot, and confocal immunofluorescence experiments. Furthermore, GnT-V down-regulation inhibited the ANP, BNP, β-MHC, and sarcomeric a-actinin expression levels in the H9C2 cells, indicating the inhibition of cardiomyocyte hypertrophy (Fig. 5B, C and D).

**Fig. 6:**
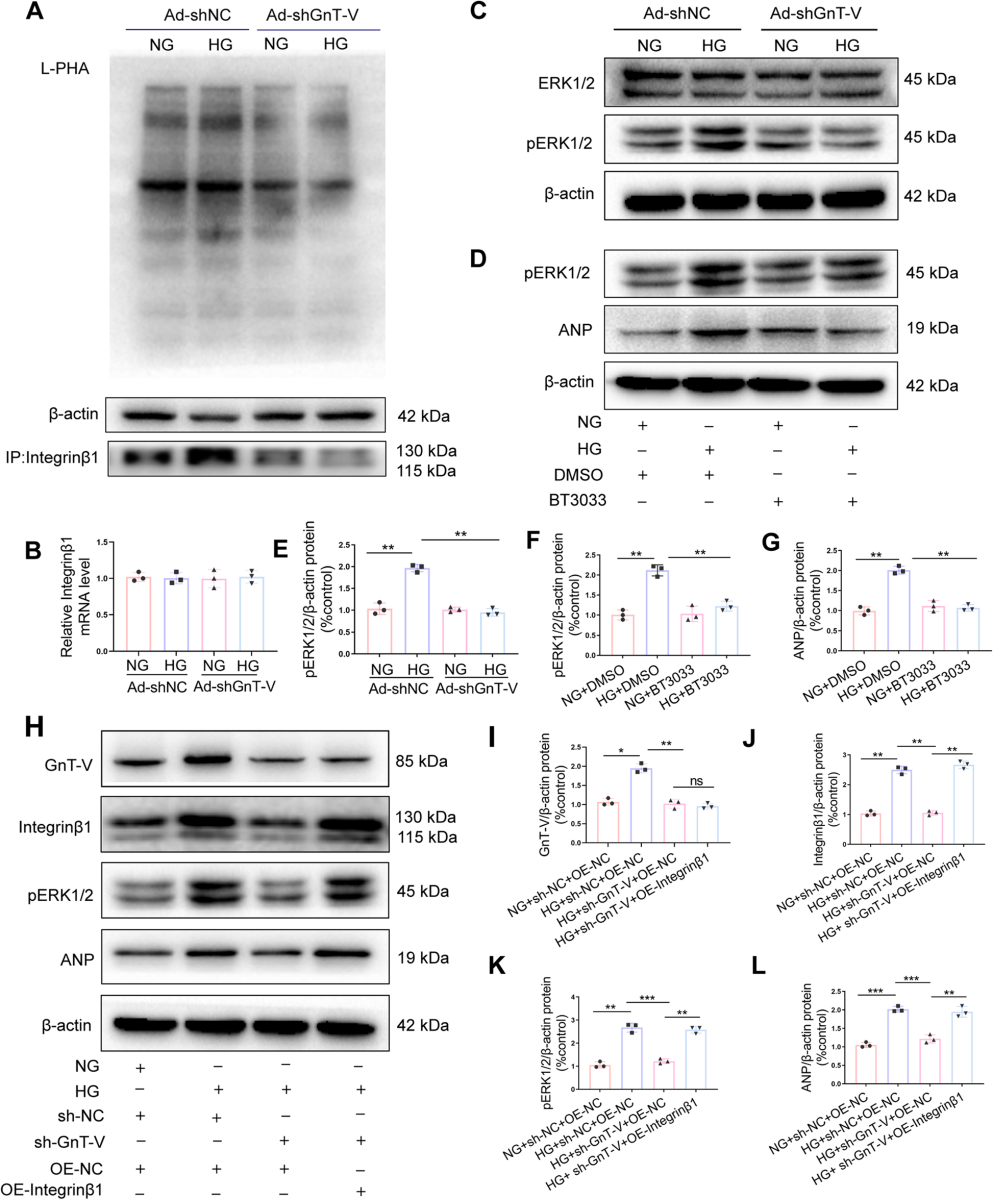
GnT-V enhanced cardiomyocyte hypertrophy via integrinβ1-ERK1/2 signaling.** A** A lectin blot analysis revealed the numbers of β-1,6-linked branches of each group. β-actin was used as a control, L-PHA precipitation (blotting with anti-Integrinβ1 staining) was performed to quantify the Integrinβ1 levels of the different groups. **B** Integrinβ1 mRNA levels were examined. **C**, **E** Western blots and quantitative assessments of phosphorylated ERK1/2 in H9C2 cells treated with shRNA against GnT-V and the control. **D**, **F**, **G** Western blots and quantitative assessments of phosphorylated ERK1/2 in H9C2 cells treated or not treated with BT-3033 (10 lmol/L; Tocris Bioscience). **H** GnT-V-knockdown H9C2 cells were transfected with an integrinβ1 overexpression plasmid, and immunoblotting was used to determine the levels of GnT, integrinβ1, phosphorylated ERK1/2, and ANP. **I**, **J**, **K**, **L** Quantitative study of integrinβ1, phosphorylated ERK1/2, and ANP protein expression level. Data presented as mean ± SD. *p < 0.05, ** < 0.01, ***p < 0.001

Previous studies have highlighted the crucial roles played by integrins and integrin-related signaling pathways in cardiac hypertrophy [8, 24, 25]. Thus, to determine if GnT-V knockdown inhibited cardiac hypertrophy in diabetic cardiomyopathy via integrin β1, we carried out in vitro experiments using H9C2 cells and an inhibitor (BTT-3033) against integrin. BTT-3033 is a selective inhibitor of integrin α_2_β_1,_ considering collagen specificity integrin α_2_ subunit is expressed only in cardiac fibroblasts but not in cardiomyocytes [26], so we examined only integrin β1. The results of the protein immunoblot experiments (Fig. 5I, J) indicated that shRNA-GnT-V transfection decreased the integrin β1 expression in HG conditions. Moreover, the amazing transfection of shRNA GnT-V in HG-treated H9C2 cells was consistent with the BTT-3033 therapy, which markedly reduced the expression of integrinβ1 and heart hypertrophic markers (Fig. 5K-N). The majority of N-glycan carriers, integrins have more than 20 possible glycosylation sites. The N-glycan core structure is necessary for integrin heterodimerization, conformation stabilization, expression at the cell membrane, and interaction with ligands in cancers [12]. To confirm whether integrinβ1 modifies with β-1,6 GlcNAc-branched structures catalyzed by the GnT-V enzyme involved in myocardial hypertrophy in diabetic cardiomyopathy model, we detected the integrinβ1 expression levels by RT-PCR. As demonstrated in Fig. 6B, the integrinβ1 expression levels in each group were invariant. Lectin blots were then performed, and the results showed that integrinβ1 galactosylated with GnT-V-mediated β-1,6 GlcNAc branches was highly elevated, accompanied by alleviated cardiac fibrosis by GnT-V expression silencing (Fig. 6A). These data indicated that integrinβ1 glycosylation could be modified by GnT-V in cardiac hypertrophy, contributing to the activation of several cardiac hypertrophy signaling pathways.

Previous research has linked the ERK1/2 cascade to cardiac hypertrophy [27]. To understand the different signaling pathways involved in diabetic cardiomyopathy, ERK activity levels in H9C2 cells with and without the transfection of shRNA GnT-V treatment were measured. As shown in Fig. 6C, E, GnT-V knockdown significantly decreased ERK 1/2 phosphorylation in H9C2 cells under HG conditions. Moreover, the results of the protein immunoblotting experiments indicated that BTT-3033 pretreatment also attenuated ERK 1/2 phosphorylation and cardiac hypertrophy (Fig. 6D, F and G). Also, when the shRNA GnT-V transfection was combined with the integrin β1 overexpression, there was an increase in the ERK 1/2 phosphorylation rate and cardiac hypertrophy marker levels in the HG-treated cells (Fig. 6H-L). Thus, these results indicated that GnT-V could regulate diabetes-induced cardiac hypertrophy via the integrin β1- ERK 1/2 signaling pathways.

### GnT-V knockdown alleviated fibrosis through TGFβ1-Smads fibrotic pathway

**Fig. 7:**
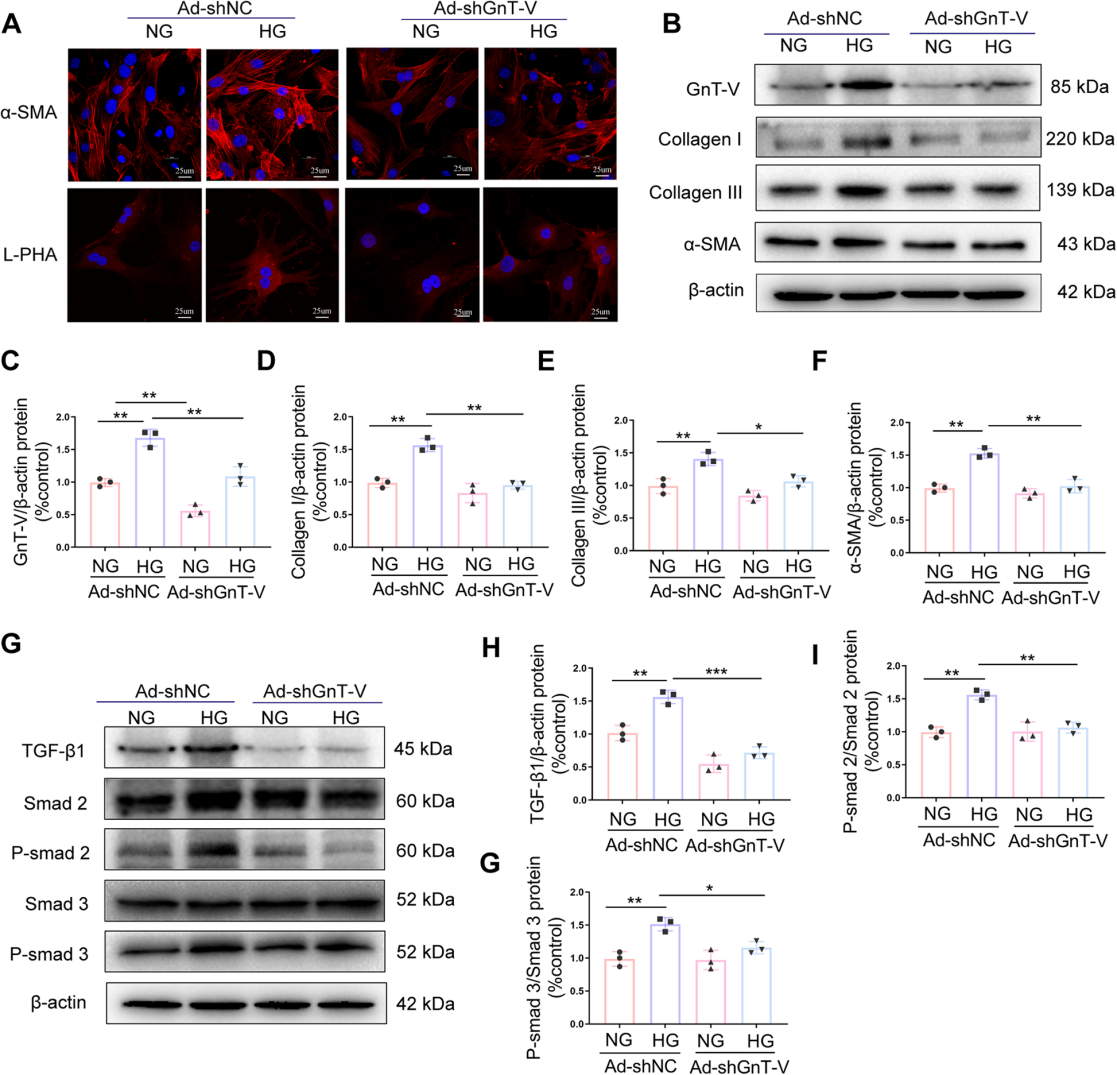
GnT-V induced heart fibrosis by activating the TGF-β1-Smads pathway. GnT-V and a negative control shRNA were transfected into HG-treated cardiac fibroblasts. **A** Confocal pictures of the proteins α-SMA and L-PHA in cardiac fibroblasts. **B**, **C**, **D**, **E**, **F** Representative immunoblots and quantitative analysis of GnT-V, CollagenI, CollagenIII, and α-SMA protein expression. **G**, **H**, **I**, **J** TGF-β1, p-Smad2, and p-Smad3 protein expression levels were quantified using immunoblots. Data presented as mean ± SD. *p < 0.05, ** < 0.01, ***p < 0.001

Further in vitro experiments were designed to explore the methods by which GnT-V knockdown affects fibrosis and to investigate the TGF1/Smads signaling pathway, which is important in mediating pathologic fibrosis and cardiac remodeling in diabetic cardiomyopathy [28, 29]. The expression of collagen I, collagen III, and α-SMA was significantly increased in the HG + Ad-shNC group, while their function was suppressed after shRNA-GnT-V transfection as shown in Fig. 7A-F. Furthermore, in comparison to the NG + Ad-shNC group, the TGF-β1/Smad2/3 pathway was either active in the HG + Ad-shNC group; however, it was significantly repressed in the HG + Ad-shGnT-V group (Fig. 7G-J). These findings indicated that the TGF-β1/Smad2/3 fibrotic pathway was responsible for the efficacy of GnT-V knockdown to alleviate fibrosis in HG-treated cardiac fibroblasts.

## Discussion

In the present study, we found that GnT-V was highly expressed in hypertrophic cardiomyocytes and myofibroblasts. Furthermore, in diabetic mice, GnT-V knockdown reduced myocardial fibrosis and hypertrophy while attenuating cardiac dysfunction. In vitro findings showed that GnT-V knockdown inhibited cardiomyocyte hypertrophy by suppressing integrinβ1 expression, leading to inhibition of downstream ERK1/2 activation, and impaired the TGF-β1/Smads signaling pathway, which further led to attenuation of cardiac fibrosis under HG conditions. Thus, the study's findings demonstrated the significance of GnT-V in diabetic cardiomyopathy and suggested that it might serve as a target molecule for the therapy of diabetic cardiomyopathy.

Metabolic glycan staining showed that N-glycosylation was elevated in db/db mice and a similar effect was observed in serum samples from patients with type 2 diabetes [30]. Additionally, GnT-V expression was found to be increased in the left ventricle of hypertensive rats [31]. In the present study, over-expression GnT-V mRNA and protein were found in cardiac tissues of diabetic mice. In addition, significant increase in cardiomyocyte size and reactivation of cardiac hypertrophy markers indicated myocardial hypertrophy. GnT-V knockdown effectively inhibited diabetes-induced myocardial hypertrophy and ameliorated cardiac function. This may be the first study to elucidate the differential expression of GnT-V in diabetic cardiomyopathy cardiomyocytes. Next, we will investigate the possible pathways regulated by GnT-V in hypertrophic cardiomyocytes in diabetes.

Integrins play an important role in cardiac physiopathology. Studies have shown that upregulation of β1, α3, and α7 integrins and redistribution of β3 integrins accompany with cardiac hypertrophy [9, 10, 32, 33]. In the present study, we found that the expression level of integrinβ1 increased paralleled to the over-expression of GnT-V in the hypertrophic cardiomyocytes stimulated by high glucose. GlcNAcβ-1,6 complex-type N-glycans synthesized by GnT-V was also increased. GnT-V knockdown significantly reduced the expression of GlcNAc β-1,6 complex-type N-glycans and integrinβ1 as well as cardiac hypertrophic response in the cardiomyocytes treated by high glucose. However, the relevant mechanisms for integrinβ1 expression regulation by GnT-V remain unclear, requiring further clarification. ERK1/2 signaling is one of canonical downstream target molecules of integrinβ1 [34]. Moreover, ERK1/2 signaling cascade implicated in the regulation of cardiomyocyte, and sustained phosphorylation of ERK1/2 appears to be detrimental to cardiac cell fate. Our results showed that ERK1/2 was obviously phosphorylated in parallel to the increased integrinβ1 expression in the cardiomyocytes treated by high glucose, and down-regulation of either GnT-V or integrinβ1 effectively inhibited phosphorylation of ERK1/2. Thus, the results demonstrated that GnT-V activated integrinβ1-REK1/2 signaling in the cardiomyocytes treated by high glucose, thereby promoting cardiomyocyte hypertrophy.

Clinical studies have shown that myocardial fibrosis is the main pathognomonic change in patients with type I or type II diabetes [35]. Transformation of myocardial fibroblasts to myofibroblasts is a critical step in the pathogenesis of myocardial fibrosis [36]. In this study, GnT-V expression was remarkably increased and severe cardiac fibrosis occurred in the diabetic heart tissues of mice. GnT-V knockdown alleviated myocardial remodeling and attenuated myocardial fibrosis in diabetic mice. In vitro data consistently demonstrated that GnT-V knockdown significantly inhibited the HG-induced activation of cardiac fibroblasts as evidenced by the downregulated expression of collagen I, collagen III and α-SMA. TGF-β1/Smads pathway is an important signaling pathway mediating the cardiac fibrosis process [28, 29]. In this study, we found that TGF-β1/Smads pathway was obviously activated in parallel to the increased GnT-V expression in HG-induced cardiac fibroblasts and down-regulation of GnT-V effectively inhibited activation of TGF-β1/Smads pathway. This result is consistent with previous studies indicating the profibrotic effect of GnT-V in scleroderma and liver cirrhosis [37–40]. Therefore, the current work offers fresh perspectives on the function and mechanism of GnT-V in diabetic cardiomyopathy and associated heart conditions.

## Conclusions

In conclusion, our findings reveal that hyperglycemia increases GnT-V expression in both cardiac myocytes and cardiac fibroblasts. In diabetic cardiomyopathy, knocking down GnT-V reduces HG-induced cardiac hypertrophy by depending on an integrinβ1-ERK1/2 dependent manner, and alleviates myocardial fibrosis by inhibiting the TGF-β1/Smads signaling pathway. These findings offer new insights into the possible mechanisms involved in cardiovascular issues related to diabetic cardiomyopathy, which could be a therapeutic target for reducing myocardial hypertrophy and fibrosis in patients with this condition.

### Supplementary Information

**Supplementary Material 1.**

**Supplementary Material 2.**

## Data Availability

No datasets were generated or analysed during the current study.

## Abbreviations

DCM: Diabetic cardiomyopathy
GnT-V: N-acetylglucosaminyltransferase V
STZ: Streptozotocin
β1,6-GlcNAc: β1,6-N-acetylglucosamine
ERK1/2: Extracellular signal-regulated kinase ERK1 and ERK2
PDGF: Platelet-derived growth factor
EGF: Epidermal growth factor
RBG: Random blood sugar
α-SMA: A-smooth muscle actin
ANP: Atrial natriuretic peptide
BNP: Brain natriuretic peptide
β-MHC: β-Myosin heavy chain
TGF-β1: Transforming growth factor β1
LVIDd: Left ventricular internal dimension in diastole
LVEF: Left ventricular ejection fraction
LVIDs: Left ventricular internal dimension in systole
LVFS: Left ventricular fractional shortening
WGA: Wheat germ agglutinin
DMEM: Dulbecco’s modified Eagle’s medium
IHC: Immunohistochemistry
FBS: Fetal bovine serum
L-PHA: Biotinylated phaseolus vulgaris leucoagglutinin
