# Supplementary material for: Inhibition of N-acetylglucosaminyltransferase V alleviates diabetic cardiomyopathy in mice by attenuating cardiac hypertrophy and fibrosis
Source: Nutr Metab (Lond). 2024 Jul 30;21:53. doi: 10.1186/s12986-024-00797-w (PMC11290217; doi:10.1186/s12986-024-00797-w)

Figure S1. Characterization of cardiac fibroblasts (CFs). Immunofluorescence analysis indicated phenotype of cardiac fibroblasts when CFs were cultured in normal glucose medium and high glucose medium. (green, Vimentin; blue, DAPI; red, MYH11). Scale bar, 50 μm.

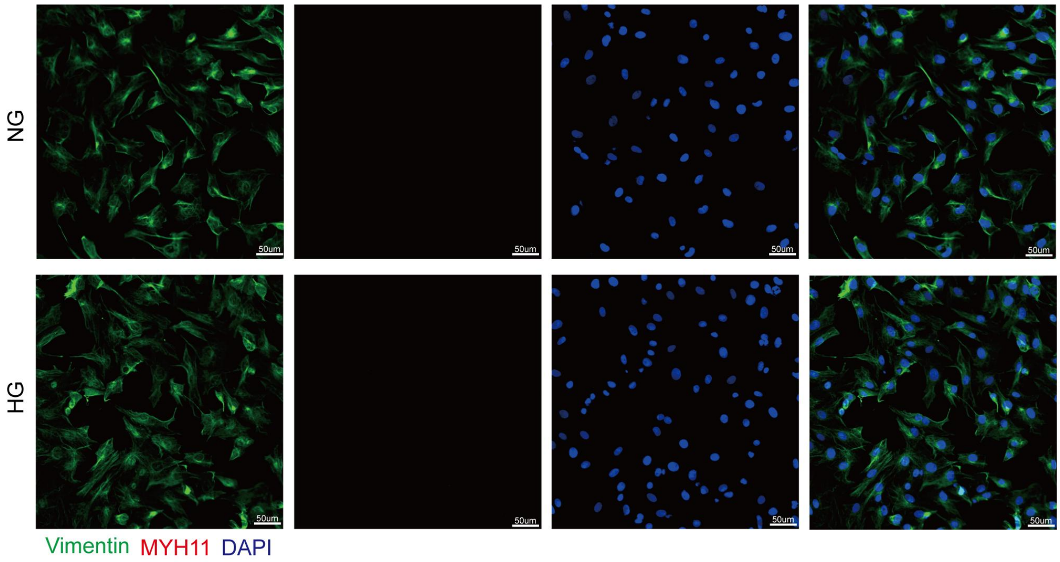

Figure S2. The expression of β-Tubulin in myocardial tissues. Western blot analysis indicated that β-Tubulin is stably expressed in myocardial tissues.

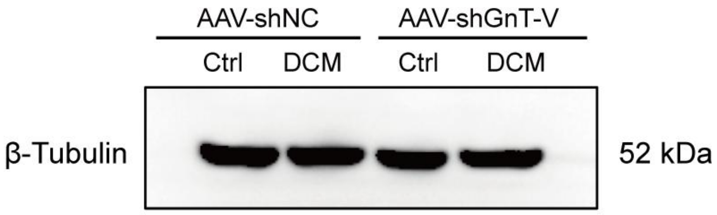

Supplement: Supplementary file 1 — Supplementary Material 1. [file 12986_2024_797_MOESM1_ESM.pdf]
