# Supplementary material for: Inhibition of N-acetylglucosaminyltransferase V alleviates diabetic cardiomyopathy in mice by attenuating cardiac hypertrophy and fibrosis
Source: Nutr Metab (Lond). 2024 Jul 30;21:53. doi: 10.1186/s12986-024-00797-w (PMC11290217; doi:10.1186/s12986-024-00797-w)
Supplement: Supplementary file 2 — Supplementary Material 2. [file 12986_2024_797_MOESM2_ESM.pdf]

Fig.S1a

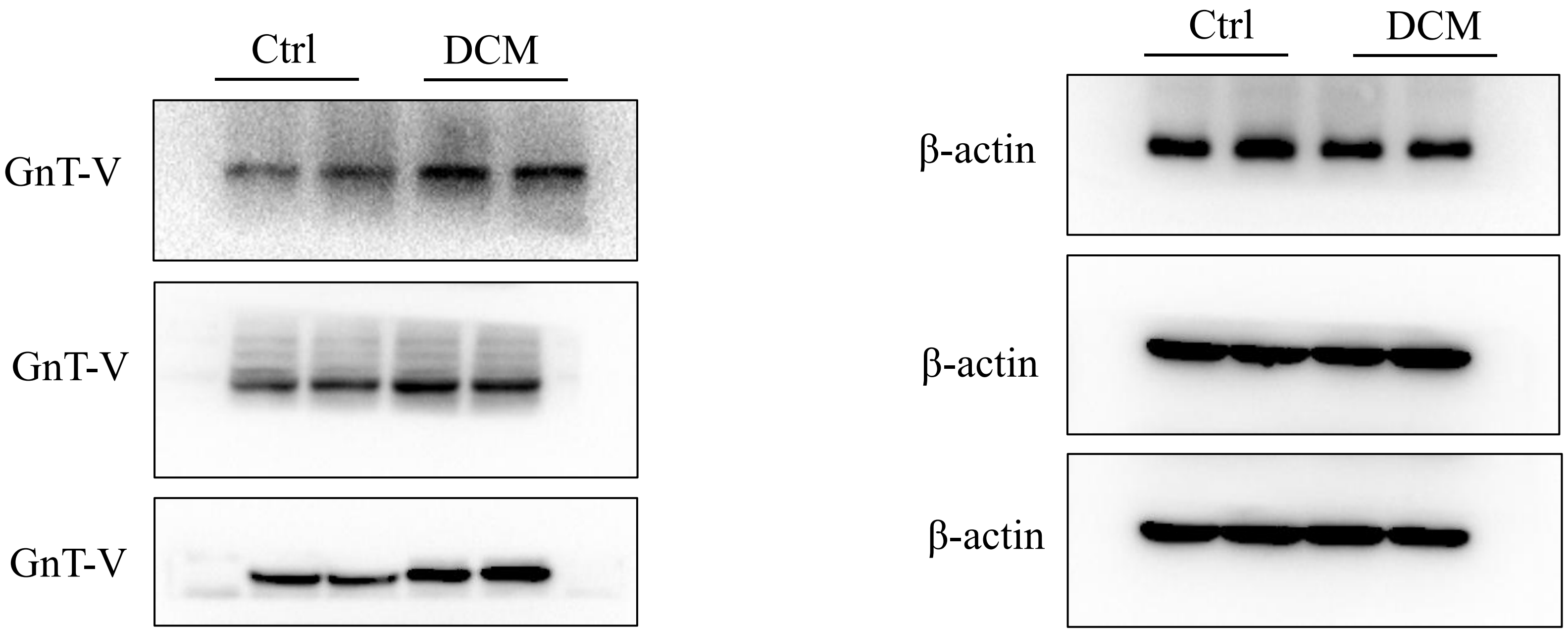

Fig.S2a

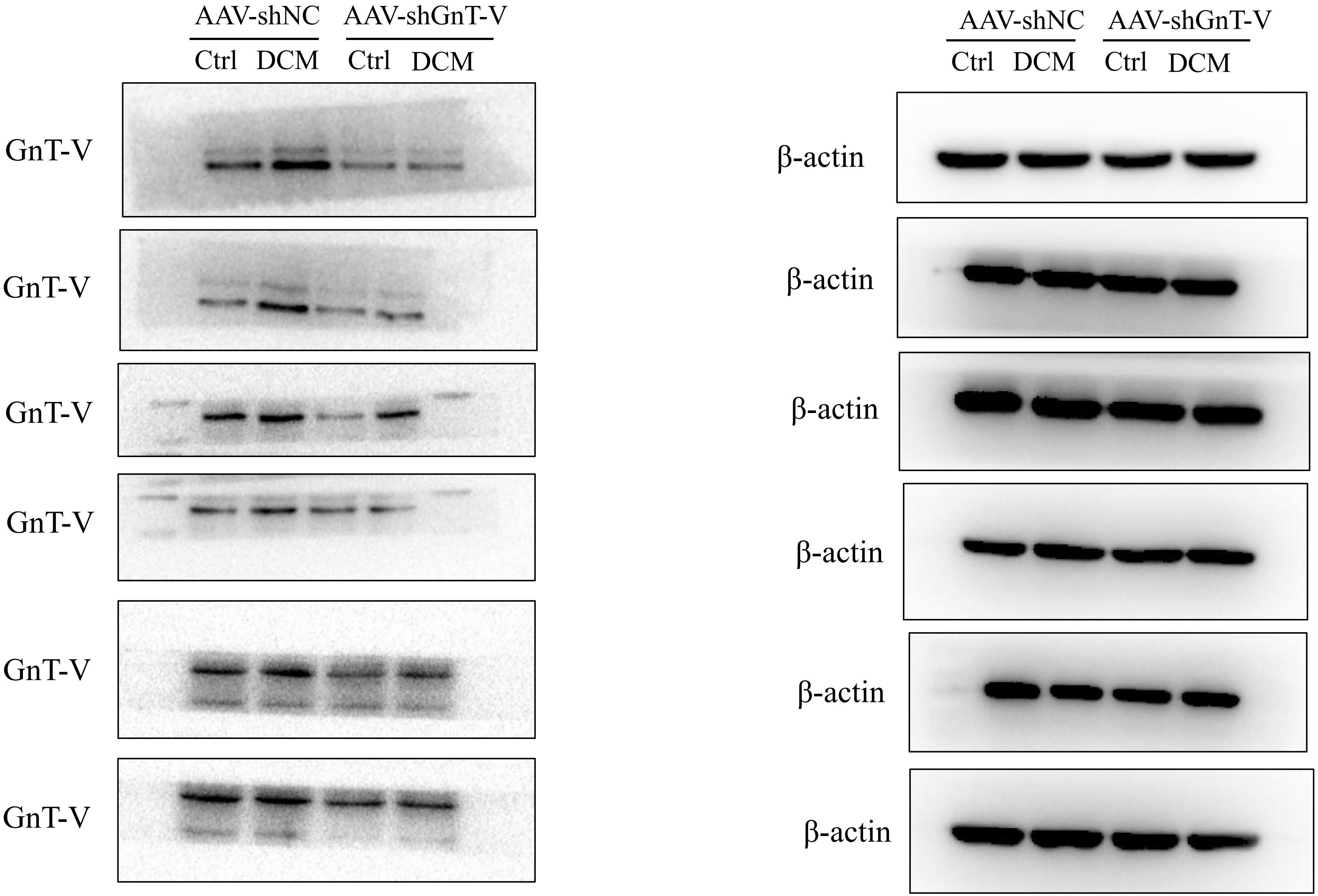
AAV-shNC
AAV-shGnT-V
Ctrl
DCM
Ctrl
DCM
GnT-V
GnT-V
GnT-V
GnT-V
GnT-V
GnT-V
AAV-shNC
AAV-shGnT-V
Ctrl
DCM
Ctrl
DCM
β-actin
β-actin
β-actin
β-actin
β-actin
β-actin

Fig.S3a

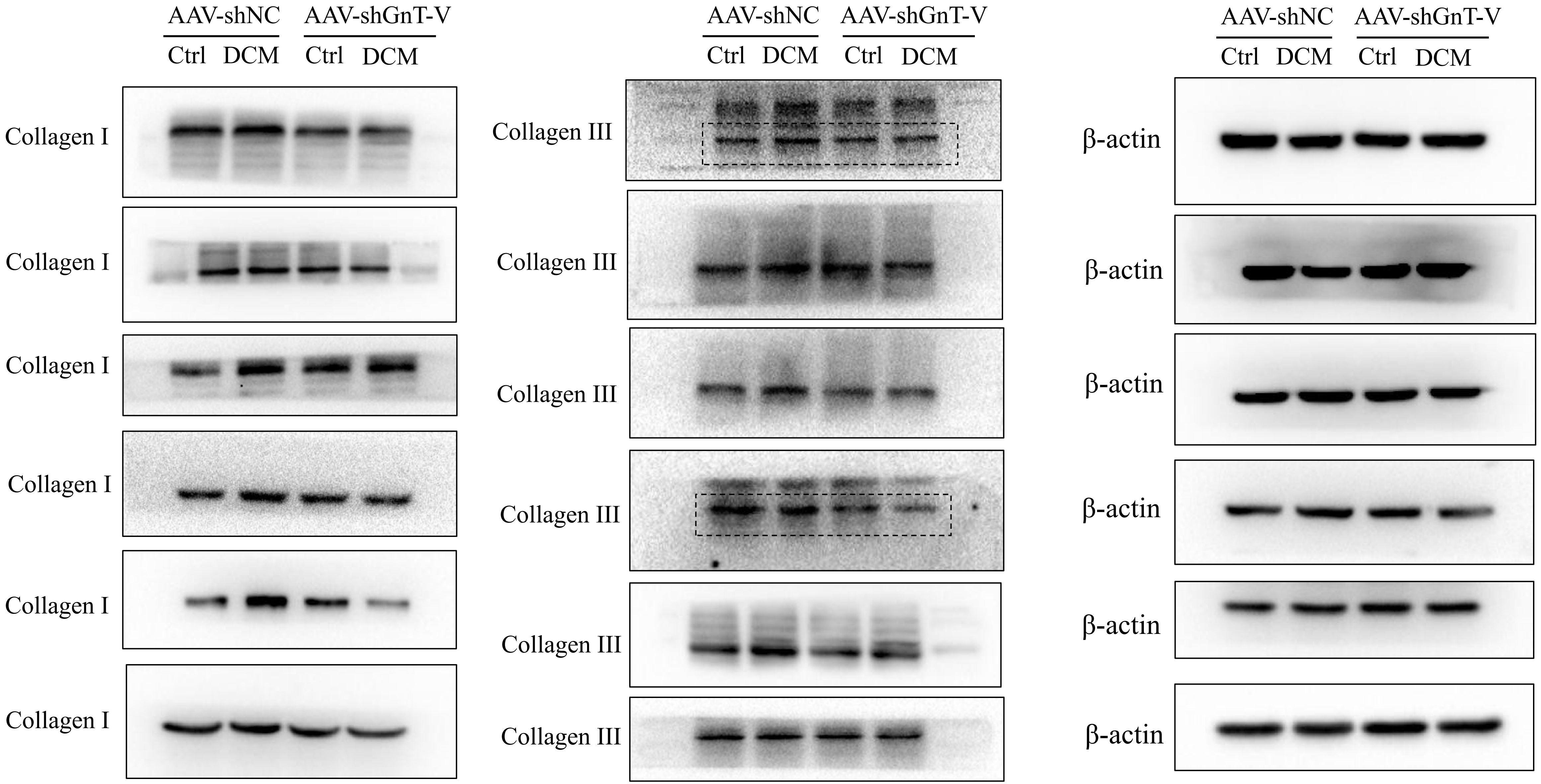

AAV-shNC
AAV-shGnT-V
Ctrl
DCM
Ctrl
DCM
Collagen I
Collagen I
Collagen I
Collagen I
Collagen I
Collagen I
Collagen III
Collagen III
Collagen III
Collagen III
Collagen III
Collagen III
β-actin
β-actin
β-actin
β-actin
β-actin
β-actin

Fig.S4a

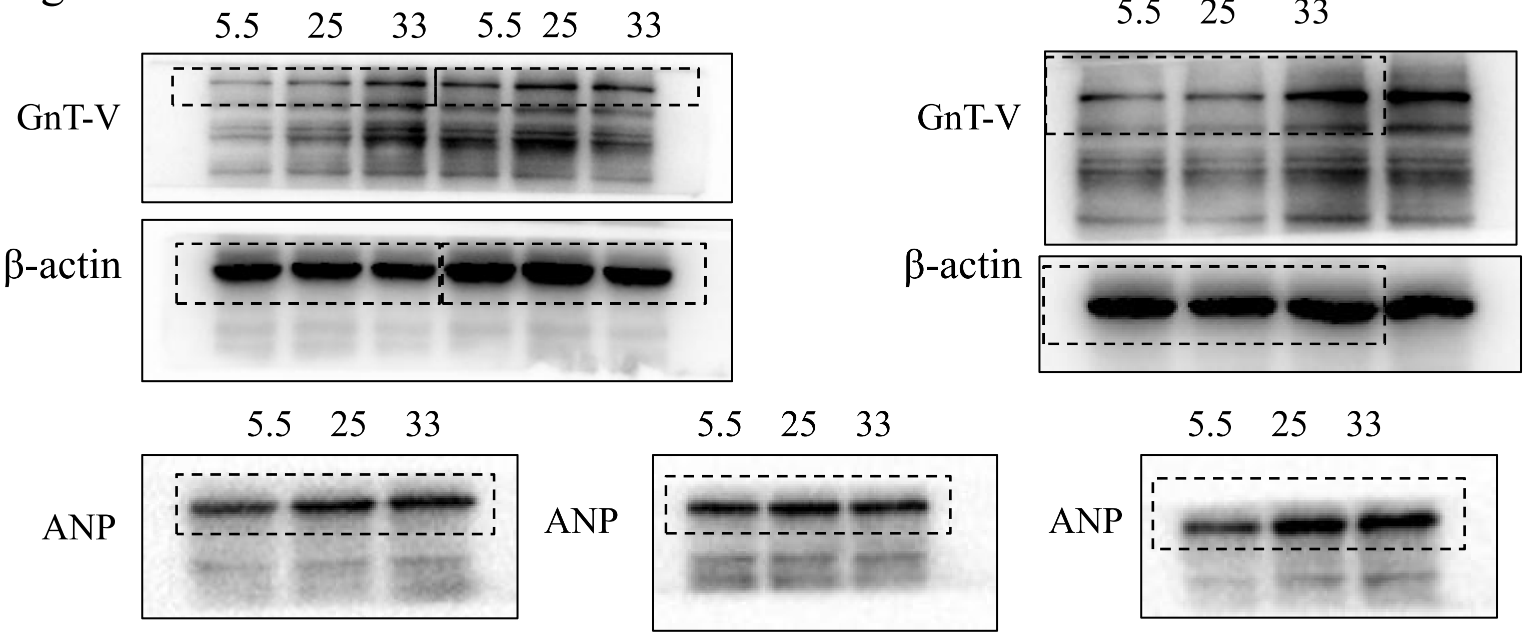

Fig.S4b

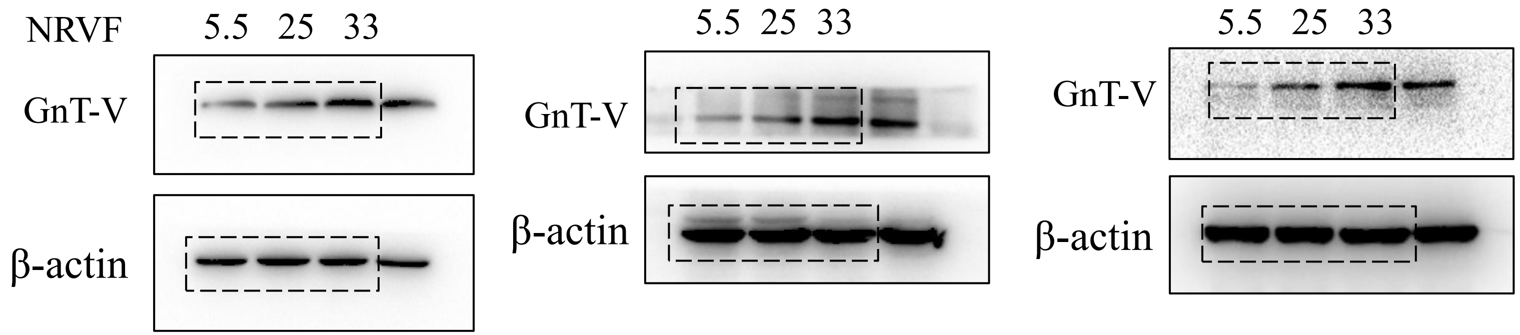

Fig.S4c

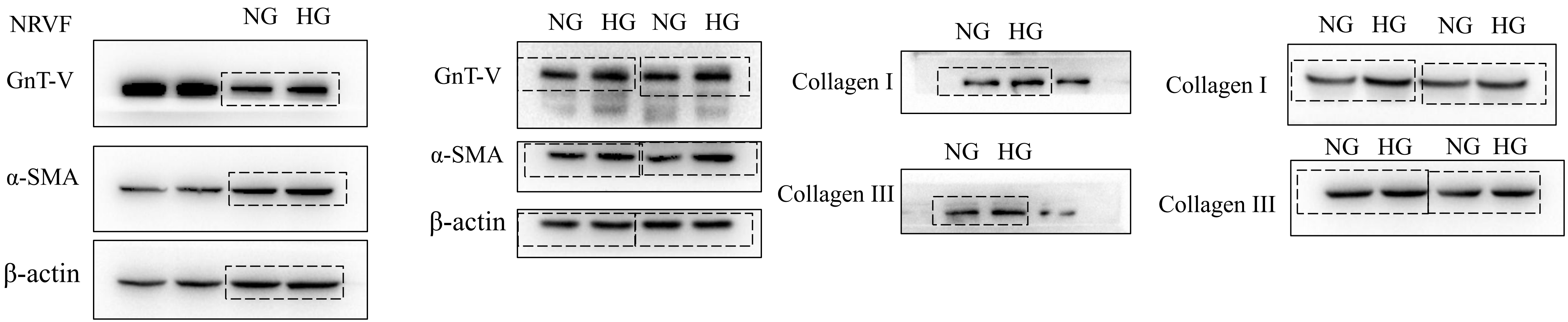
NRVF
NG HG
GnT-V
α-SMA
β-actin
NG HG NG HG
GnT-V
α-SMA
β-actin
NG HG
Collagen I
NG HG
Collagen III
NG HG NG HG
Collagen I
NG HG NG HG
Collagen III

Fig.S5a

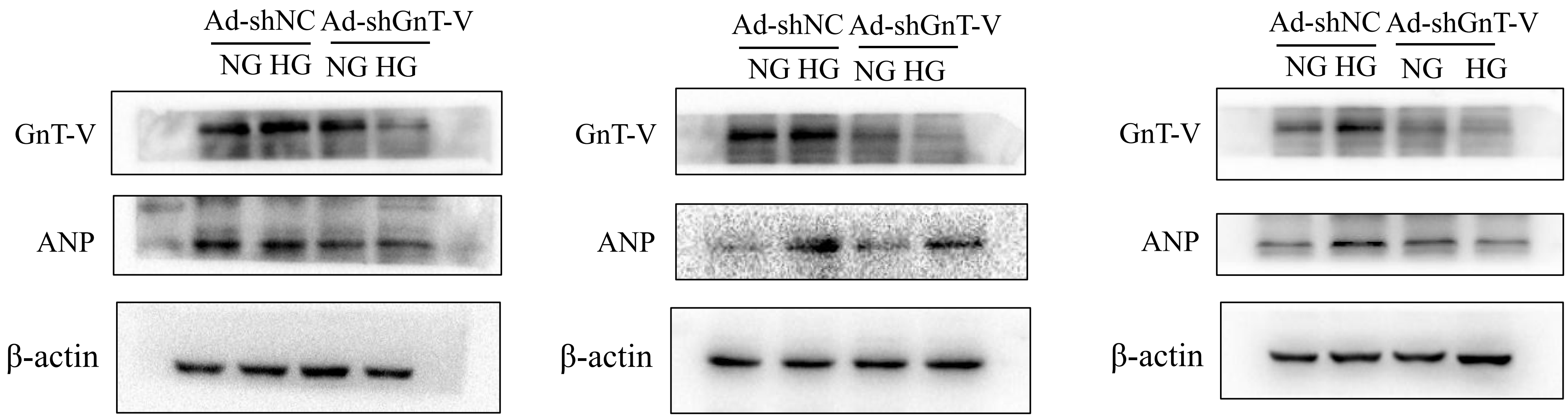

Fig.S5b

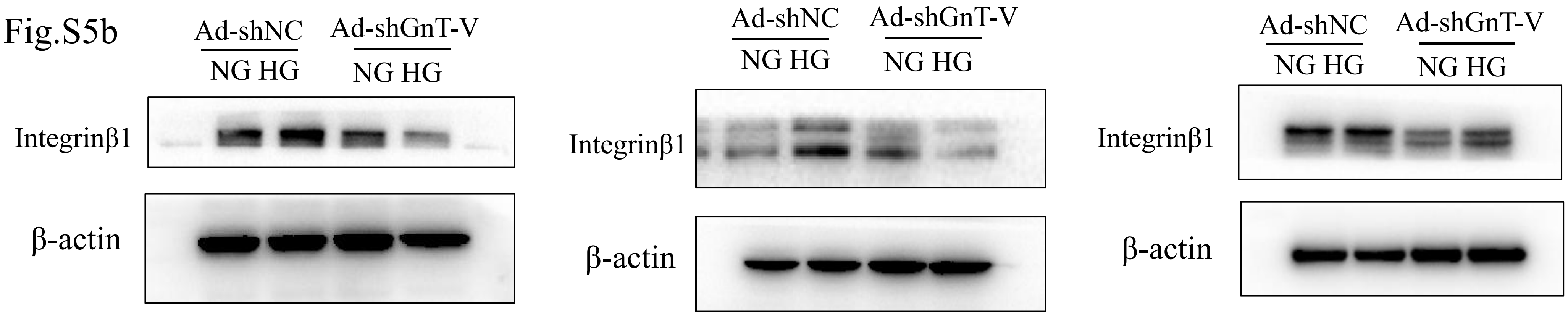

Fig.S5c

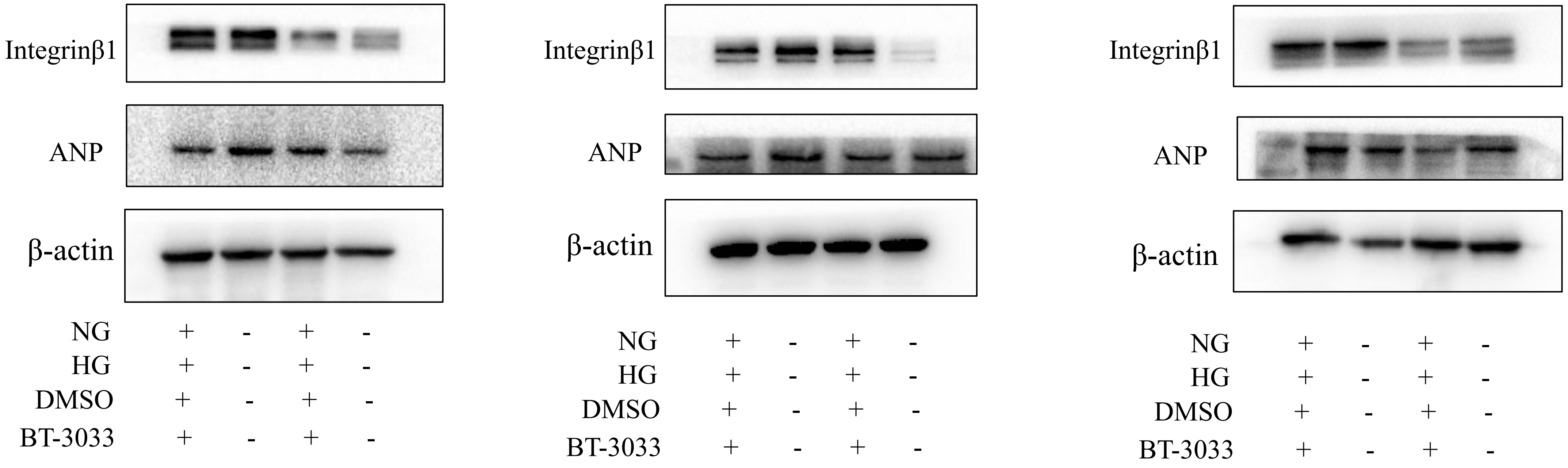
Integrinβ1
ANP
β-actin
NG + - + -
HG + - + -
DMSO + - + -
BT-3033 + - + -
Integrinβ1
ANP
β-actin
NG + - + -
HG + - + -
DMSO + - + -
BT-3033 + - + -
Integrinβ1
ANP
β-actin
NG + - + -
HG + - + -
DMSO + - + -
BT-3033 + - + -

Fig.S6a

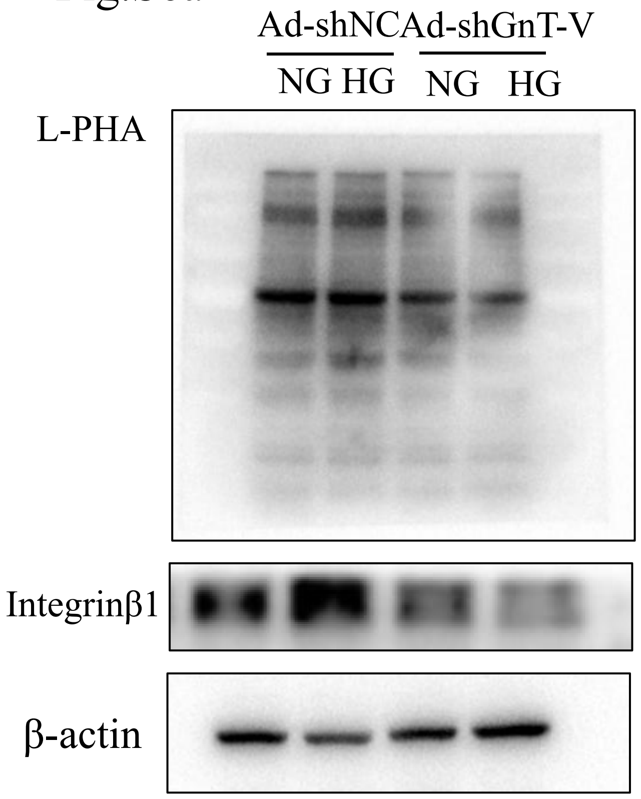

Fig.S6b

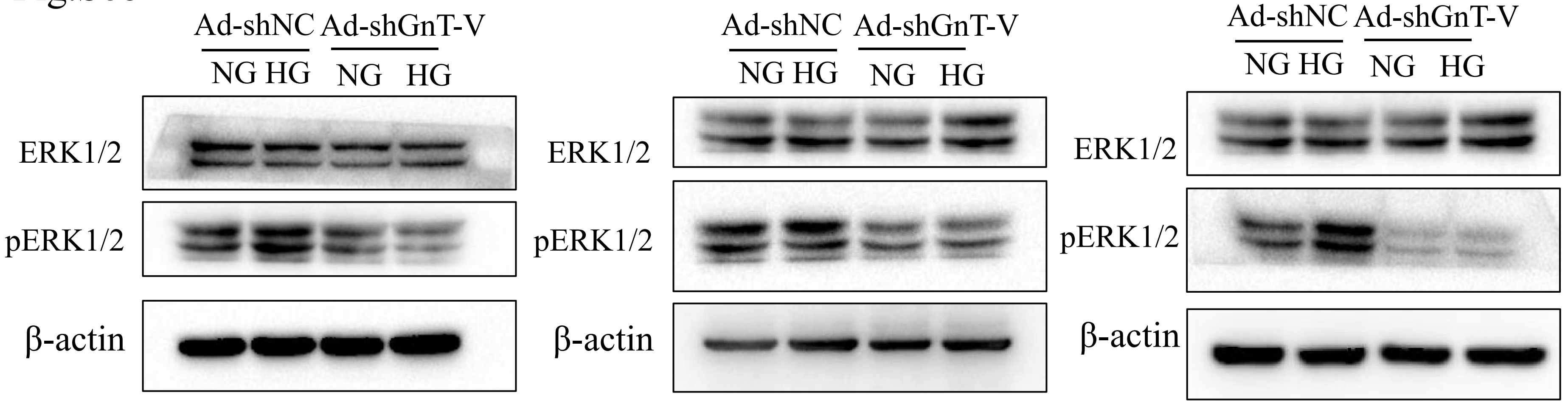

Fig.S6c

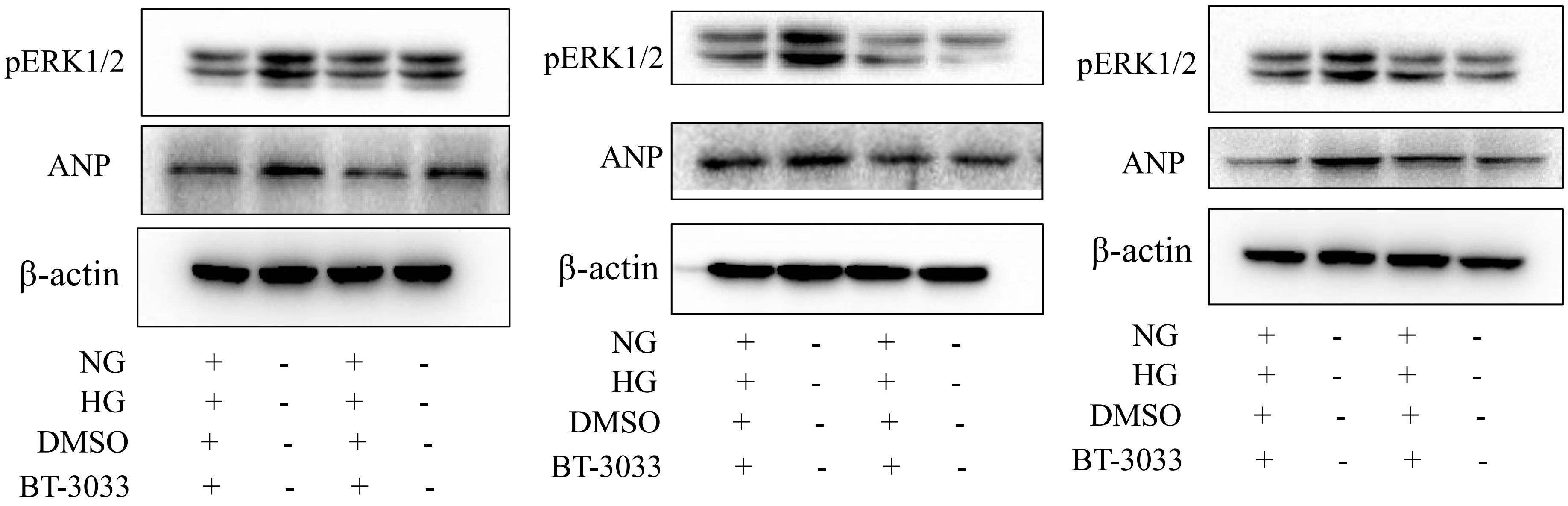

Fig.S6d

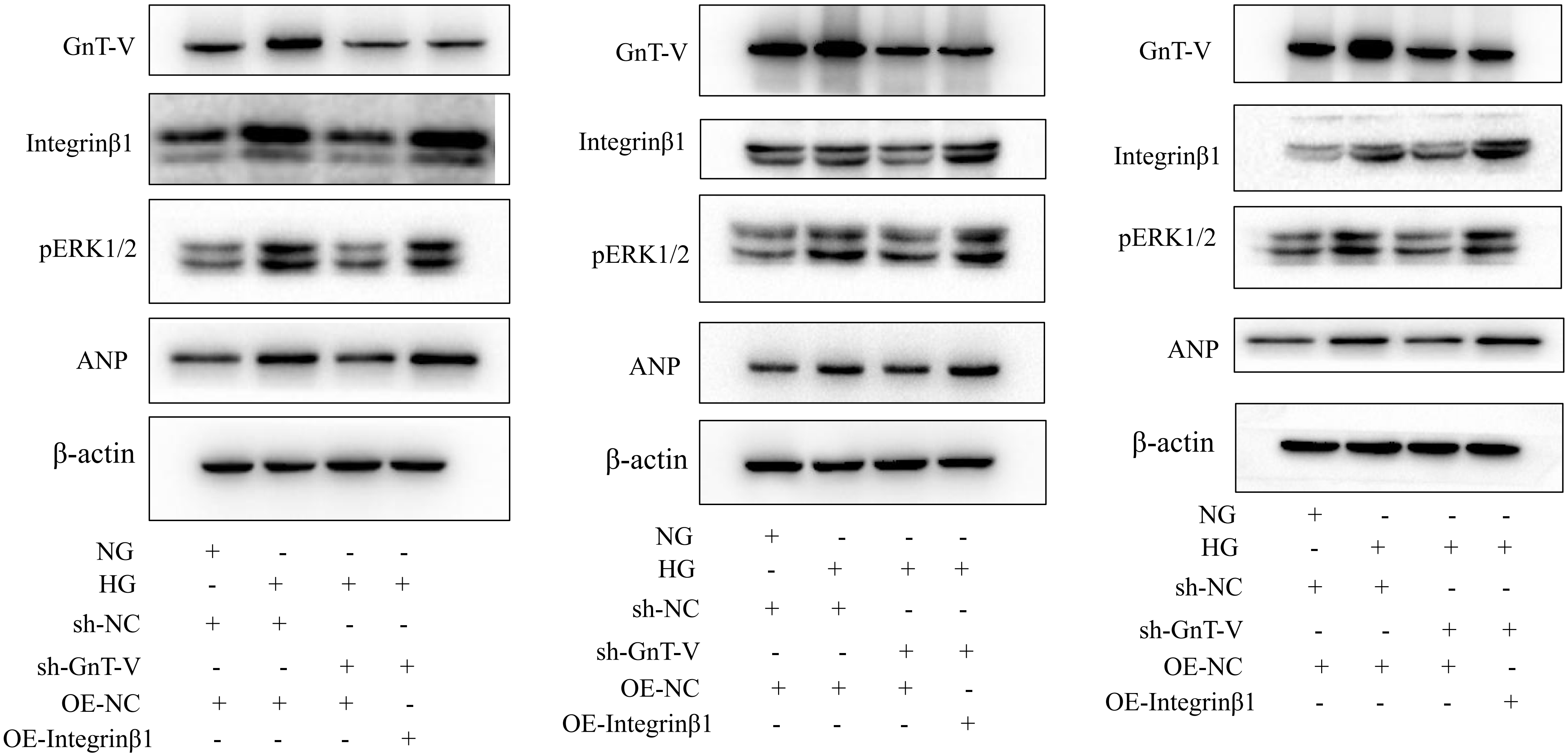
GnT-V
Integrinβ1
pERK1/2
ANP
β-actin
NG + - - -
HG - + + +
sh-NC + + - -
sh-GnT-V - - + +
OE-NC + + + -
OE-Integrinβ1 - - - +
GnT-V
Integrinβ1
pERK1/2
ANP
β-actin
NG + - - -
HG - + + +
sh-NC + + - -
sh-GnT-V - - + +
OE-NC + + + -
OE-Integrinβ1 - - - +
GnT-V
Integrinβ1
pERK1/2
ANP
β-actin
NG + - - -
HG - + + +
sh-NC + + - -
sh-GnT-V - - + +
OE-NC + + + -
OE-Integrinβ1 - - - +

Fig.S7a

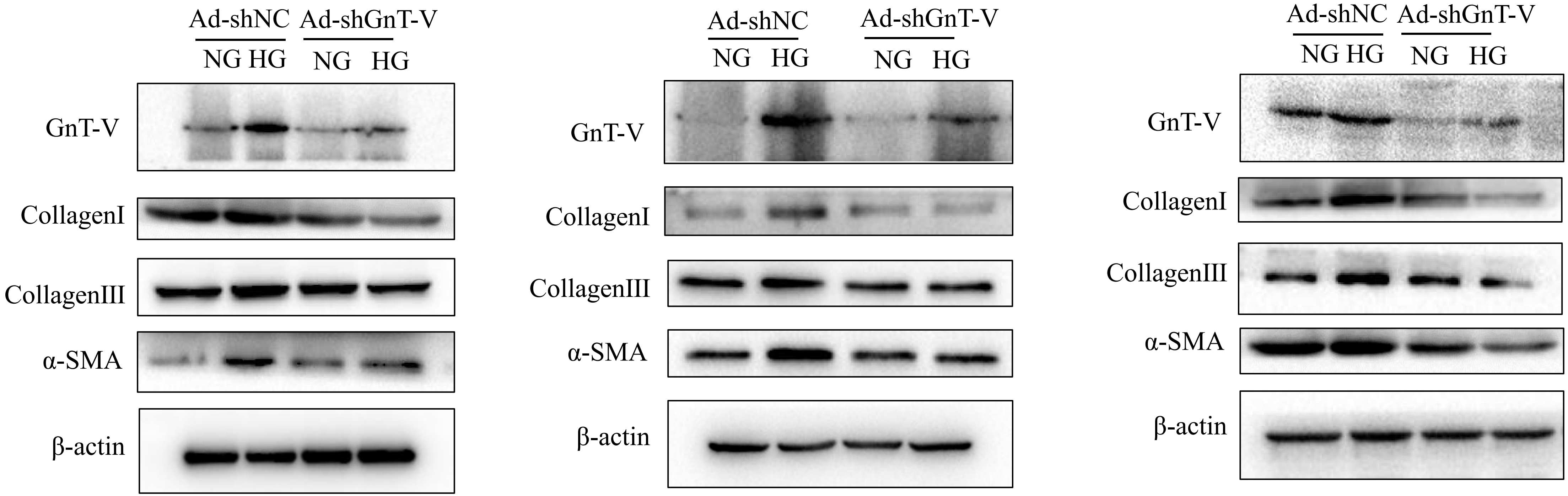
Ad-shNC
Ad-shGnT-V
NG
HG
NG
HG
GnT-V
CollagenI
CollagenIII
α-SMA
β-actin
Ad-shNC
Ad-shGnT-V
NG
HG
NG
HG
GnT-V
CollagenI
CollagenIII
α-SMA
β-actin
Ad-shNC
Ad-shGnT-V
NG
HG
NG
HG
GnT-V
CollagenI
CollagenIII
α-SMA
β-actin

Fig.S7b

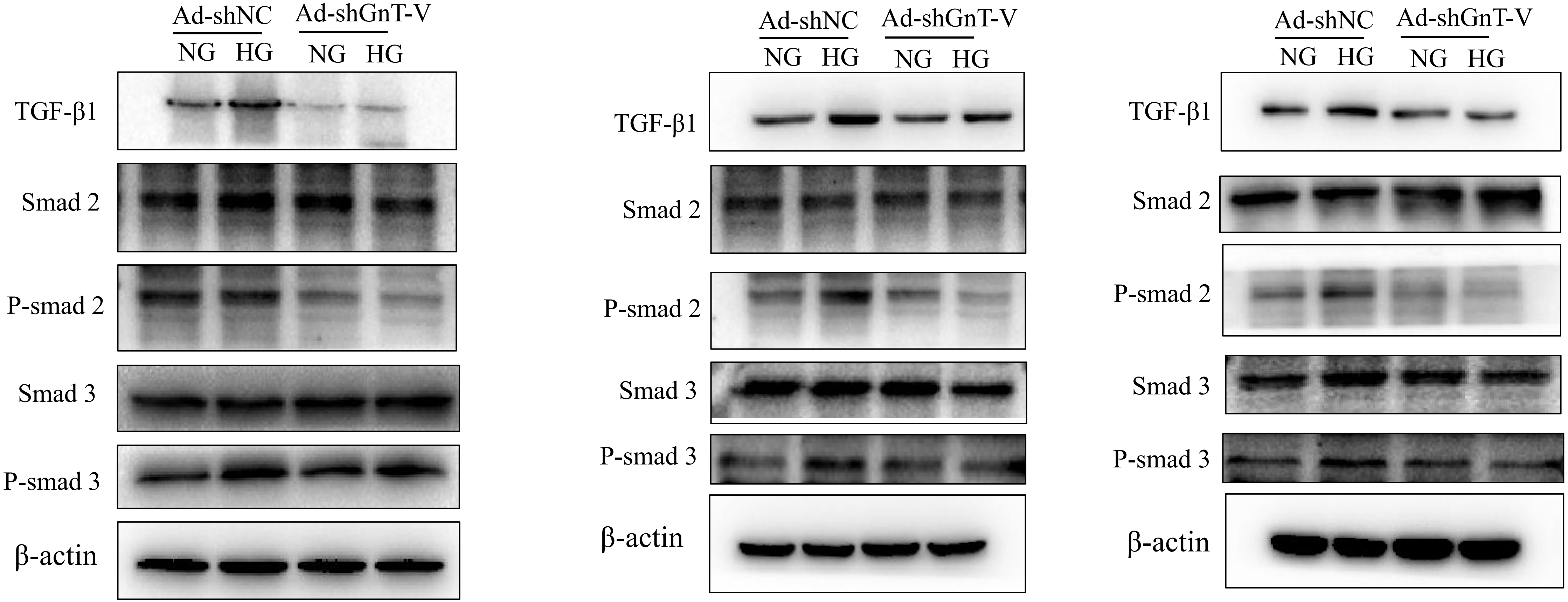
Ad-shNC
Ad-shGnT-V
NG
HG
NG
HG
TGF-β1
Smad 2
P-smad 2
Smad 3
P-smad 3
β-actin
